# Artificial Intelligence for Automated Recognition of Hepatocystic Anatomy During Laparoscopic Cholecystectomy: Current Evidence, Clinical Readiness, and Future Directions

**DOI:** 10.3390/medicina62091705

**Published:** 2026-09-05

**Authors:** Catalin Dumitru Cosma, Dragos Calin Molnar, Marian Botoncea, Cosmin Nicolescu, Calin Molnar, Vlad-Olimpiu Butiurca

**Affiliations:** 1 Faculty of Medicine, George Emil Palade University of Medicine, Pharmacy, Sciences and Technology of Târgu Mureș, 540139 Targu Mures, Romania; catalin.cosma@umfst.ro (C.D.C.); marian.botoncea@umfst.ro (M.B.); cosmin.nicolescu@umfst.ro (C.N.); calin.molnar@umfst.ro (C.M.); vladbutiurca@yahoo.com (V.-O.B.); 2 General Surgery Clinic No. 1, County Emergency Clinical Hospital of Târgu-Mureș, 540136 Targu Mures, Romania

**Keywords:** artificial intelligence, computer vision, laparoscopic cholecystectomy, hepatocystic triangle, critical view of safety, bile duct injury, surgical safety, deep learning, intraoperative guidance

## Abstract

*Background and Objectives:* Bile duct injury remains a major safety concern during laparoscopic cholecystectomy, and reliable interpretation of hepatocystic anatomy is fundamental to safe dissection. Artificial intelligence (AI)-based computer vision may support anatomical recognition, critical view of safety (CVS) assessment, and intraoperative decision support. This narrative review synthesized the current evidence, clinical applications, readiness for implementation, and future requirements of anatomy-aware AI during laparoscopic cholecystectomy. *Materials and Methods:* Five bibliographic databases were searched through 15 August 2026, supplemented by citation tracking and targeted searches. Studies were classified according to clinical task, dataset, reference standard, validation design, performance metrics, real-time capability, human factor assessment, and clinical readiness stage. The evidence base comprised 105 verified references: 50 primary AI reports and 55 contextual or methodological sources; 39 direct model development or evaluation reports were characterized in detail. *Results:* Investigated applications included landmark detection, semantic segmentation, CVS assessment, safe and hazard zone mapping, multimodal analysis incorporating indocyanine green fluorescence, automated documentation, education, and real-time perceptual prompting. Among the 39 direct reports, 24 remained at the offline proof of concept or internal validation stage, eight achieved temporal, external, or multicenter validation, six demonstrated prospective operating-room feasibility, and one reached post-deployment surveillance. The latter evaluated a surgical-process outcome rather than patient morbidity. Generalizability was constrained by dataset overlap, heterogeneous reference standards and metrics, domain shift, and underrepresentation of difficult cholecystectomy. No included study demonstrated reduced bile duct injury or other patient-level benefit. *Conclusions:* Despite progression to prospective feasibility and one post-deployment process surveillance report, no included study demonstrated a reduction in bile duct injury or another patient-level outcome. Current evidence supports adjunctive applications in documentation, video triage, education, coaching, and quality assurance, while surgeon-facing deployment requires further multicenter, human factor, and comparative-effectiveness evaluation.

## 1. Introduction

Laparoscopic cholecystectomy (LC) is the standard operative treatment for symptomatic gallstone disease and one of the most frequently performed procedures in general surgery. Its favorable recovery profile, however, coexists with a small but clinically consequential risk of bile duct injury (BDI). Reported incidence varies according to case mix and outcome definition: contemporary large-series estimates place major BDI at approximately 0.15–0.36%, whereas broader estimates encompassing less severe biliary injuries and bile leaks reach 0.4–1.5%. Given the global volume of cholecystectomy, even these low percentages translate into a substantial absolute burden. BDI may result in biliary peritonitis, sepsis, benign biliary stricture, recurrent cholangitis, repeated endoscopic, radiological or surgical interventions, secondary hepatic injury, impaired long-term quality of life, and excess mortality. Prevention therefore remains a defining patient safety objective in biliary surgery [1,2,3,4].

Major BDI is commonly a problem of anatomical misidentification rather than technical imprecision alone. In a landmark human factor analysis of 252 laparoscopic BDIs, a visual perceptual illusion was identified as the primary cause in 97% of cases, whereas an isolated deficit in technical skill accounted for only a small minority [5]. The archetypal error is the misidentification of the common bile duct or common hepatic duct as the cystic duct, followed by dissection and transection within an incorrect anatomical model. Acute or chronic inflammation, fibrosis, a contracted gallbladder, an impacted stone at the gallbladder neck, anatomical variation, bleeding, smoke, excessive traction, and an unfavorable camera perspective can further distort familiar visual cues. Consequently, the central safety challenge is not merely knowledge of normal anatomy but reliable recognition of surgically relevant anatomy under dynamic and frequently adverse operative conditions [6,7,8].

The term “Calot’s triangle” historically refers to the region bounded by the cystic duct, common hepatic duct, and cystic artery, whereas the hepatocystic triangle used in contemporary safe cholecystectomy practice is bounded superiorly by the inferior surface of the liver. Throughout this review, “hepatocystic triangle” denotes the modern operative dissection field, while “Calot’s triangle” is retained only in historical or quoted contexts. The critical view of safety requires clearance of this triangle, separation of the lower third of the gallbladder from the cystic plate, and confirmation that two and only two structures enter the gallbladder [3,4,6,7,9,10].

Despite its conceptual clarity and broad endorsement, the CVS is not uniformly achieved, interpreted, or documented in routine practice. Video- and image-based studies have demonstrated incomplete attainment, inconsistent documentation, and clinically meaningful variability in human assessment, particularly regarding clearance of the hepatocystic triangle [11,12,13,14,15]. Moreover, severe inflammation and operative difficulty may make complete CVS attainment unsafe or impossible [8,16]. This creates two related gaps: an implementation gap between a recommended safety method and its consistent execution, and a verification gap between the surgeon’s intraoperative judgment and an objective, reproducible record of what was actually achieved. These limitations are also relevant to AI development because the visual reference standard used for model training is itself vulnerable to interobserver disagreement, incomplete visualization, and label noise.

The native video stream generated during LC makes the procedure particularly suited to computer vision (CV), the branch of artificial intelligence (AI) that extracts clinically meaningful information from images and temporal sequences. Early image guidance work established the feasibility of computer-assisted preparation of Calot’s triangle [17], while subsequent deep learning studies demonstrated automated recognition or segmentation of anatomical landmarks and hepatocystic structures [18,19,20]. Later research extended these capabilities to localization and assessment of the CVS [21,22,23], delineation of relatively safe and hazardous operative regions [24,25], and prospective or operating-room-ready real-time deployment [26,27,28]. In principle, such systems could function as continuous perceptual adjuncts by highlighting critical anatomy, prompting a CVS time-out, warning when dissection approaches a high-risk region, facilitating structured documentation, and supporting education or postoperative quality review [29,30,31].

Nevertheless, promising model performance does not establish clinical readiness. Much of the published evidence has been generated from retrospectively selected frames or videos, frequently obtained from single institutions and characterized by heterogeneous target definitions, annotation protocols, units of analysis, test strategies, and performance metrics [32,33,34,35]. External and multicenter validation remains less common than internal testing, while domain shift may arise from differences in patient anatomy, inflammatory severity, operative technique, camera systems, image compression, smoke, blood, tissue deformation, and partial occlusion. Real-time clinical use introduces additional requirements, including low-latency inference, calibration, uncertainty communication, failure detection, ergonomic display design, workflow compatibility, and safeguards against automation bias or alarm fatigue [35,36,37,38]. Accordingly, this review distinguishes algorithmic performance, real-time feasibility, human factor and operative-process effects, and patient-level effectiveness as separate evidentiary domains.

Several reviews have already characterized this rapidly evolving field. A 2024 narrative review summarized broad AI applications across operative phase recognition, anatomy, and cholecystitis [39]; systematic reviews published in 2025 focused more specifically on automated anatomical structure and landmark recognition [32,33]; and a 2026 review examined AI-assisted safety assessment [34]. The most recent comprehensive systematic review identified 85 primary CV studies in LC through March 2026, including 14 studies of anatomy recognition or segmentation and 14 studies of safety assessment, while emphasizing heterogeneous reporting, limited external validation, and persistent implementation barriers [35]. A complementary, clinically anchored synthesis is therefore warranted to connect anatomical targets and model outputs with the actual decision points of safe LC, incorporate the newest multimodal and real-time evidence, and appraise readiness for clinical use rather than technical performance alone.

Throughout this review, AI is considered an adjunct to surgical expertise and established safety principles rather than an autonomous substitute for operative judgment, and a clear distinction is maintained between anatomical recognition, safety-process assessment, and demonstrated improvement in patient outcomes [37,40,41].

## 2. Materials and Methods

### 2.1. Review Design and Scope

This article was designed as a structured narrative review with critical synthesis. Its preparation was informed by the Scale for the Assessment of Narrative Review Articles (SANRA), particularly its requirements concerning justification of the review objective, transparency of the literature search, appropriate referencing, scientific reasoning, and presentation of relevant endpoint data [42]. The review was not conceived as a systematic review or meta-analysis, and no protocol was registered. Nevertheless, a prespecified and reproducible search, selection, and evidence classification process was applied to reduce arbitrary study selection and explicitly define the boundaries of the evidence base.

The principal review question was as follows: What evidence supports artificial intelligence (AI)-assisted recognition of hepatocystic anatomy and safety-critical operative states during laparoscopic cholecystectomy (LC), and how close are these systems to clinically meaningful implementation? The core scope comprised computer vision methods that identified, localized, classified, detected, or segmented relevant anatomical structures; evaluated achievement of the critical view of safety (CVS); delineated relatively safe or hazardous dissection regions; or integrated anatomy-aware information into real-time guidance, documentation, quality assessment, or education. Studies addressing adjacent tasks, including surgical phase, instrument, or action recognition, were considered only when their findings were explicitly connected to anatomical interpretation, CVS assessment, dissection safety, contextual scene understanding, or intraoperative deployment. Clinical anatomy, BDI prevention, surgical safety, fluorescence imaging, methodological, reporting, and regulatory literature were used to establish the clinical and translational context but were not classified as direct primary AI evidence.

This article was classified as a structured narrative review because its purpose was the critical interpretation of a focused clinical–translational question rather than the broad mapping of concepts, evidence types, and knowledge gaps that primarily characterizes a scoping review. The prespecified search, selection, and extraction procedures were used to improve transparency and reduce arbitrary study selection but were not intended to confer systematic review or scoping review status.

### 2.2. Information Sources and Search Strategy

Structured searches covered PubMed/MEDLINE, Embase, Scopus, Web of Science Core Collection, and IEEE Xplore from database inception to 15 August 2026. The strategy combined controlled vocabulary, where available, with title, abstract, and keyword terms describing AI, LC, hepatocystic anatomy, and safety-related operative interpretation. Database-specific syntax, truncation symbols, and field tags were adapted to the indexing structure of each information source. No outcome or performance metric filter was applied because terminology, task definitions, validation strategies, and reported endpoints remain heterogeneous across this emerging field.

**The core search concept was:**

(“artificial intelligence” OR “machine learning” OR “deep learning” OR “computer vision” OR “neural network*” OR “convolutional neural network*” OR “semantic segmentation” OR “instance segmentation” OR “object detection” OR “image recognition”) AND (“Cholecystectomy, Laparoscopic” OR “laparoscopic cholecystectom*” OR “laparoscopic gallbladder surgery”).

The broad AI/procedure search was subsequently combined with the following complementary anatomy and safety block:

(Calot* OR hepatocystic OR “critical view of safety” OR CVS OR “bile duct*” OR landmark* OR anatom* OR “safe zone*” OR “hazard zone*” OR dissection OR navigation).

Separate targeted searches were undertaken to identify authoritative publications concerning hepatocystic anatomy, CVS, BDI prevention, operative difficulty assessment, fluorescence cholangiography, surgical AI reporting standards, validation metrics, human–AI interaction, and staged translational evaluation. The reference lists of relevant reviews, eligible primary studies, dataset reports, guidelines, and consensus documents were examined, and forward citation tracking was used to identify linked, updated, or subsequent studies. Google Scholar and preprint servers were used only as supplementary discovery tools. Preprints were retained only when they provided uniquely informative descriptions of datasets, benchmarks, emerging human–machine interfaces, or future technical directions. They were explicitly identified as non-peer-reviewed evidence and were not treated as established clinical-effectiveness data.

### 2.3. Eligibility Criteria

Primary studies were included when they fulfilled the following criteria: (i) they analyzed human LC images or videos, or an LC dataset derived from human procedures; (ii) they developed, validated, or evaluated an AI or computer vision method directly addressing anatomical structure recognition, localization, detection, segmentation, CVS identification or assessment, safe/hazard zone mapping, anatomy-aware dissection guidance, AI-assisted fluorescence interpretation, or real-time anatomy-related support; and (iii) they provided sufficient methodological and quantitative information to characterize the intended task, dataset, reference standard, validation strategy, and reported performance. Peer-reviewed full-length journal articles, peer-reviewed conference proceedings containing adequate methodological information, and dataset or benchmark reports were considered. Reviews, consensus statements, clinical guidelines, and seminal clinical anatomy publications were retained for contextual and interpretive synthesis.

Studies were excluded when they addressed perioperative risk prediction, administrative outcomes, pathology, or preoperative radiology without intraoperative scene interpretation; recognized workflow phases or instruments without an explicit anatomy-, CVS-, dissection-, or safety-related application; evaluated conventional fluorescence imaging or image enhancement without an AI component; or were editorials, letters, or conference abstracts without a complete report, duplicate publications, or reports with insufficient methodological detail. When several publications described overlapping datasets, successive versions of the same system, or different evaluations arising from a common research program, the most complete peer-reviewed report was prioritized for the relevant technical question, while scientifically distinct companion reports were retained and linked to avoid inappropriate double counting. No language restriction was applied during database retrieval; however, inclusion required availability of a full text that could be assessed reliably in English.

### 2.4. Study Selection and Reference-Pool Construction

Retrieved records were imported into a reference-management workspace and deduplicated using DOI, PMID, title, author names, publication year, and study characteristics. Titles and abstracts were independently assessed by two reviewers, followed by full-text evaluation of potentially relevant reports. Disagreements were resolved through discussion and, when required, adjudication by a senior reviewer. A single principal reason for exclusion was assigned during full-text assessment. Related conference and journal publications, corrections, dataset reports, external validation studies, and updated versions were cross-referenced to establish publication and dataset lineage.

After final deduplication, citation tracking, metadata verification, and classification, the evidence base comprised 105 source records. These consisted of 50 primary AI records and 55 clinical, anatomical, methodological, reporting, regulatory, or translational sources. Among the 50 primary AI records, 39 directly developed, validated, prospectively deployed, or evaluated human interaction with an anatomy- or safety-oriented model. The remaining 11 primary reports supplied companion evidence concerning historical image guidance, adjacent workflow analysis, surgical-scene reasoning, documentation, coaching, education, deployment, and emerging multimodal approaches.

**Figure 1:**
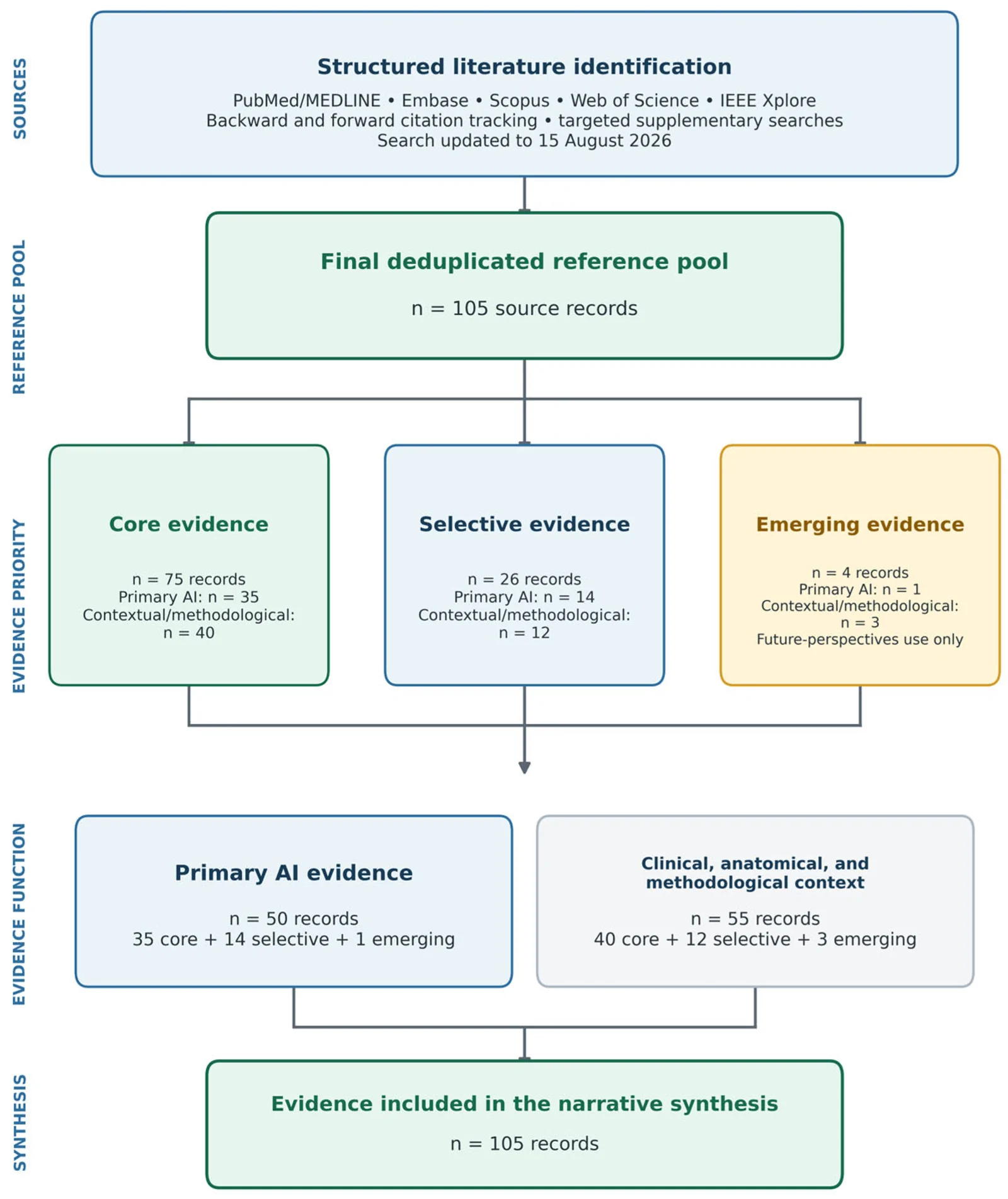
Study identification and composition of the final evidence base. The final deduplicated pool comprised 105 records: 50 primary AI reports and 55 clinical, anatomical, methodological, reporting, regulatory, or translational sources. According to their role in the synthesis, 75 records were classified as core evidence, 26 as selective supporting evidence, and four as emerging evidence.

According to their role within the synthesis, 75 records were classified as core evidence, 26 as selective supporting evidence, and four as emerging evidence. This relevance-based classification was used to control the evidentiary role assigned to each publication and was not interpreted as a formal risk of bias or methodological quality score. Emerging reports were used primarily to define future research directions and were explicitly distinguished from peer-reviewed clinical evidence. The identification process and composition of the final evidence base are summarized in Figure 1.

### 2.5. Data Extraction and Evidence Classification

A structured extraction framework was developed specifically for this review. For each primary AI study, the following information was recorded where available: bibliographic details; country and number of participating centers; retrospective or prospective design; dataset provenance; number of procedures, patients, videos, images, clips, and frames; unit of allocation to development, validation, and test sets; target anatomy or safety construct; AI task and model architecture; annotation process; reference standard definition; number and expertise of annotators; adjudication procedures; internal, temporal, external, or multicenter validation; class-specific and aggregate performance metrics; processing speed or latency; real-time deployment; user-interface characteristics; prospective human use; effects on surgeon interpretation or operative behavior; operative-process measures; patient-level outcomes; funding and conflicts of interest; and availability of data, annotations, model weights, and source code.

The extracted evidence was organized into six clinically oriented task groups: (1) landmark or anatomical structure recognition; (2) pixel-level semantic or instance segmentation; (3) CVS localization, component recognition, or achievement assessment; (4) safe/hazard zone mapping and dissection guidance; (5) multimodal or indocyanine-green-assisted recognition; and (6) real-time deployment, automated documentation, education, and human factor evaluation. These task groups were not considered mutually exclusive because individual systems could combine anatomical segmentation, temporal interpretation, safety assessment, and intraoperative deployment.

The level at which each result was calculated—frame, image, clip, video, procedure, surgeon, or patient—was preserved during extraction. Class-specific values were distinguished from macro-averaged, micro-averaged, weighted, or overall metrics. Performance values were also interpreted according to the reference standard definition, class prevalence, decision threshold, aggregation method, and validation setting. This distinction was necessary because numerically similar estimates may represent materially different clinical and technical constructs.

### 2.6. Structured Domain-Based Methodological Appraisal and Clinical Readiness Classification

The eligible evidence encompassed semantic and instance segmentation studies, object detection and classification studies, dataset reports, retrospective model development studies, external validation studies, prospective feasibility evaluations, human factor experiments, and post-deployment analyses. Because established risk of bias instruments are design-specific, no single instrument could provide a valid and comparable assessment across all included study types. We therefore did not assign standardized study-level categories such as low, moderate, or high risk of bias. Instead, a prespecified, structured domain-based methodological appraisal was performed. This appraisal was descriptive and should not be interpreted as a validated formal risk of bias assessment or numerical quality score.

The appraisal domains were informed by problem-aware recommendations for biomedical image analysis validation, DECIDE-AI, the 2024 Checklist for Artificial Intelligence in Medical Imaging, STARD-AI, and the IDEAL-D framework [38,40,43,44,45,46]. These sources were used as methodological guidance rather than as interchangeable retrospective compliance checklists, particularly because several publications preceded the relevant AI-specific reporting standards.

The following methodological domains were examined: clinical and technical representativeness of the dataset; clarity and reproducibility of the annotation and reference standard; number and expertise of annotators; adjudication of disagreement; independence of training, validation, and test data; protection against patient-, procedure-, video-, or frame-level leakage; appropriateness of metrics for the intended task; handling of class imbalance, missing data, uncertainty, calibration, and failure cases; external, temporal, and multicenter validation; computational feasibility and latency; robustness to differences in operative technique, camera systems, smoke, blood, occlusion, inflammation, fibrosis, and atypical anatomy; usability and human factors; prospective clinical evaluation; evidence of changes in surgeon cognition, behavior, or operative processes; patient-level safety and effectiveness outcomes; and transparency regarding datasets, code, funding, and conflicts of interest.

For clinical interpretation, each of the 39 direct model development or evaluation reports was assigned the highest translational stage demonstrated in the publication: Stage 0, offline proof of concept without a clearly independent procedure-level evaluation; Stage 1, internal held-out or cross-validated evaluation with explicit separation at the appropriate patient or procedure level; Stage 2, temporal, external, or multicenter validation; Stage 3, prospective real-time feasibility, shadow-mode deployment, or operating-room human factor evaluation; Stage 4A, real-world post-deployment surveillance of technical reliability, human interaction, or operative-process outcomes without comparator-controlled patient-level effectiveness; and Stage 4B, comparative clinical-effectiveness evidence demonstrating patient-level safety or benefit. Stage 4B represented the highest evidentiary level. This classification constituted a review-specific interpretive synthesis rather than a regulatory designation, validated numerical score, or assessment of product approval.

### 2.7. Data Synthesis

Findings were synthesized narratively according to AI task, intended clinical function, dataset provenance, validation setting, methodological characteristics, and clinical readiness stage. Meta-analysis was not undertaken because of substantial heterogeneity in anatomical targets, annotation definitions, dataset composition, data partitioning strategies, units of analysis, performance metrics, decision thresholds, and validation designs. Reported values were transcribed at the level and using the aggregation method reported in the original publication. Values were not mathematically pooled, converted, or ranked unless definitions and denominators were directly compatible.

When multiple performance estimates were available, priority was given to clinically relevant class-specific results and held-out, temporal, external, or multicenter test performance rather than training set results or maximal cross-validation estimates. Models were not ranked solely according to their highest reported accuracy, intersection-over-union, Dice coefficient, sensitivity, specificity, average precision, mean average precision, or area under the receiver-operating-characteristic curve. Interpretation emphasized whether the evaluation reflected the intended clinical function, whether the reference standard was credible, whether data leakage was adequately excluded, whether uncertainty and failure modes were examined, and whether performance was reproduced outside the development environment.

Technical performance, real-time feasibility, effects on surgeon interpretation or behavior, operative-process changes, and patient-level outcomes were treated as distinct levels of evidence. A technically accurate model was not assumed to be clinically effective, and an absence of reported adverse events was not interpreted as evidence of safety. When multiple publications arose from overlapping datasets or research programs, their findings were described as scientifically related rather than as independent replications.

Detailed study-level extraction was retained in Supplementary Table S1, whereas the main-text evidence table was organized by AI function and clinical readiness stage to facilitate clinically oriented interpretation.

### 2.8. Ethical Considerations

This review used published studies and publicly available bibliographic information and did not involve the collection, linkage, or reanalysis of identifiable patient-level data. Institutional review board approval and individual informed consent were therefore not required for the conduct of the review. Ethical approval, informed consent procedures, data governance, video anonymization, and data-sharing provisions reported by individual primary studies were considered when relevant to dataset provenance and clinical deployment.

## 3. Results

### 3.1. Composition, Scope, and Dependence of the Evidence Base

To improve clinical interpretability, Table 1 summarizes the principal AI functions, maturity of the supporting evidence, potential current applications, major failure risks, and recommendations regarding clinical deployment. The complete study-level evidence matrix, including study design, dataset characteristics, validation strategy, principal performance estimates, and assigned clinical readiness stage for all 39 direct reports, is provided in Supplementary Table S1. The remaining 11 primary reports provided companion evidence concerning datasets, historical image guidance, adjacent workflow applications, coaching, education, documentation, deployment, and emerging multimodal methods. An adjacent workflow study that segmented operative phases, steps, and tasks was retained because contextual state recognition may support future anatomy-aware assistance [47]. The direct evidence accumulated rapidly between 2021 and 15 August 2026, but its growth was driven primarily by retrospective image and video analyses and iterative work within a limited number of research programs rather than by independent comparative clinical trials.

The apparent number of publications overstates the number of independent data-generating programs. The Oita/J-SUMMIT family included successive landmark recognition, prospective feasibility, human factor, scar detection, and bleeding-robustness reports [18,23,26,54,62,63,65,66]. The CAMMA/EndoDigest/Endoscapes family included automated CVS clip extraction, CVS criterion recognition, operating-room deployment, spatial reasoning, temporal modeling, weakly supervised learning, and foundation-model extensions [21,22,28,48,67,68,69,70]. GoNoGoNet was developed, externally tested, and subsequently applied retrospectively to BDI videos [19,24,25]. SurgSmart was reported in a multicenter quality-control study and a later post-deployment implementation analysis [49,50]. These publications addressed different research questions and should not be treated as duplicates; however, they do not constitute statistically or scientifically independent replications.

Study designs, anatomical targets, annotation standards, case mix, data partitioning strategies, and reporting metrics were markedly heterogeneous. Some studies evaluated individual frames, whereas others generated predictions over images, short clips, full videos, complete procedures, or surgeon decisions. Aggregate accuracy, class-specific sensitivity, intersection-over-union (IoU), Dice/F1 score, average precision (AP), mean average precision (mAP), temporal localization error, user ratings, and operative-process scores therefore represented different constructs. Consequently, quantitative pooling or rank ordering of models was not considered methodologically defensible.

No included study reported a comparator-controlled reduction in bile duct injury, morbidity, conversion, reintervention, length of stay, or another patient-level outcome; the evaluated endpoints were predominantly technical performance, real-time feasibility, surgeon behavior, documentation quality, or operative-process measures.

### 3.2. Taxonomy of AI Tasks and Intended Clinical Functions

Six clinically oriented task groups were identified (Table 2). Landmark and structure detection systems localized discrete targets such as the common bile duct, cystic duct, gallbladder, liver segment IV edge, or Rouvière’s sulcus. Pixel-level methods delineated visible anatomy, the hepatocystic triangle, connective or scar tissue, or predefined operative regions. CVS systems ranged from temporal localization of a documentation window to independent recognition of the three CVS criteria and procedure-level classification of CVS achievement. Safe/hazard zone models generated spatial prompts intended to discourage unsafe dissection. Multimodal systems incorporated indocyanine-green (ICG) fluorescence or synthetic representations of visually degraded scenes. Finally, deployment and human factor studies investigated real-time operation, shadow-mode integration, automated documentation, education, coaching, or changes in surgeon interpretation.

These task groups were clinically related but not interchangeable. Detecting visible anatomy does not establish the course of an occult duct; segmenting a putative safe region does not authorize tissue division; recognizing one CVS component does not prove that the complete CVS has been achieved; and extracting a documentation clip does not establish that the dissection itself was safe. The intended decision point, display mode, unit of prediction, and clinical consequence of error therefore remained essential to interpretation.

### 3.3. Landmark Recognition and Anatomical Segmentation

Early landmark-navigation work demonstrated technical feasibility but also highlighted the difficulty of detecting small biliary structures. In the first YOLOv3 report, AP was 0.320 for the common bile duct and 0.074 for the cystic duct, compared with 0.314 for the liver segment IV edge and 0.101 for Rouvière’s sulcus. Nevertheless, two expert surgeons considered the displayed landmarks valid in 22 of 23 test videos [18]. The discrepancy between relatively low object detection AP and favorable observer acceptability illustrates that clinician ratings, spatial overlap, and temporal usefulness measure different properties. The development dataset also excluded bleeding and marked fibrosis or scarring, limiting inference for the adverse operative conditions in which anatomical assistance may be most valuable.

Subsequent systems reported higher internal performance for larger landmarks or composite anatomical regions. A YOLOv7 system detecting Rouvière’s sulcus and the liver segment IV base achieved an mAP of 91.3% with a processing time of 0.179 s/image, and surgeons accepted its derived guidance line in 95.71% of relevant images [56]. Internal segmentation studies reported mean IoU values of 74.65% [20] and 85.5% [74]. Semi-supervised segmentation of the cystic duct and cystic artery reached an IoU of 65%, close to the reported inter-annotator IoU of 70% [55]. These estimates were not directly comparable because the studies differed in anatomical targets, denominators, split strategies, annotation uncertainty, and aggregation methods.

A particularly informative counterexample was provided by a feature-pyramid-network model that achieved an internal mean IoU of 0.95896 following a random image-level split of CholecSeg8k but produced substantial false-negative segmentation when qualitatively applied to unlabeled private images [64]. This finding demonstrated that exceptional within-dataset performance can coexist with poor domain transfer. Similarly, relation-aware detection improved benchmark mAP, although absolute AP50–95 remained below 25% for the cystic plate, cystic artery, and cystic duct across the evaluated models [72]. A lightweight semi-supervised detector trained using only three labeled source videos achieved an mAP50 of approximately 70% but retained limited labeled-video diversity [71]. Robotic dual-task learning produced strong internal segmentation, with a mean IoU of 83.9%, and CVS classification, with an F1 score of 78%; however, modality shift and reuse of public benchmarks constrain direct extrapolation to conventional LC [75]. Conference reports described additional attention-based and boundary-fuzziness approaches, although complete denominators and independently verifiable performance information were limited [73,76].

### 3.4. Safe/Hazard Zone Mapping and Dissection Guidance

Go/No-Go mapping was initially developed using a geographically diverse retrospective frame set. Internal cross-validation yielded F1 scores of 0.70 for Go zones and 0.83 for No-Go zones [19]. During prospective external validation, the corresponding F1 scores decreased to 0.58 and 0.80, respectively, and Go-zone sensitivity was only 0.52 despite an overall accuracy of 0.92 [24]. The lower sensitivity outside the development environment and the asymmetry between Go- and No-Go-zone performance emphasize that high overall accuracy may be dominated by background pixels and that omission and overextension have different clinical consequences.

Retrospective application to 11 BDI and 11 control videos provided construct evidence: injury cases contained more sharp, blunt, and total instrument interactions outside the model-defined Go zone [25]. However, injury-causing interactions were distributed across No-Go, Go, and unclassified regions, and the analysis did not evaluate whether displaying the model prospectively would have altered the causal sequence. The findings therefore support an association between unsafe operative behavior and model-defined regions rather than demonstrating BDI prevention.

More recent region-mapping systems extended the concept to single-incision LC and incision planning. A two-institution Mask2Former study reported F1 scores of 0.966 for safe regions and 0.923 for hazard regions on held-out videos [57]. A separate alert-zone system preserved an IoU of approximately 0.70 against external expert ground truth and, in a randomized video-based pilot experiment, increased correct safe-incision selection from 58% to 90% [58]. In an offline interpretation experiment, anatomy overlays changed 26.9% of surgeon responses, of which 70% were subsequently judged to represent safer decisions [65]. These human factor signals are clinically relevant but were generated outside live comparative surgery. Across this task group, the model output is best interpreted as a perceptual or cognitive prompt: it cannot certify the absence of an occult duct, replace adequate exposure and traction, or convert an unsafe operative field into one suitable for tissue division.

### 3.5. CVS Localization, Documentation, and Achievement Assessment

Automated documentation represented one of the earliest mature CVS applications. In 100 held-out EndoDigest videos, cystic duct division was localized with a mean absolute error of 62.8 s, and the extracted 2.5 min clip permitted CVS assessment in 91% of cases [21]. In a subsequent four-center cohort, the mean localization error increased to 124.0 s, and only 75.0% of 144 automatically extracted clips documented the CVS effectively [48]. This multicenter decrement is clinically important: temporal localization may reduce the burden of video review, but an automatically selected clip must still be examined for completeness and cannot independently establish that all CVS criteria were achieved.

Frame- and video-level CVS recognition produced moderate internal performance. DeepCVS reported an mAP of 71.9% and balanced accuracy of 71.4% for the three CVS criteria [22]. An attention-based multiple instance learning approach reported a mean unweighted accuracy of 82.6% for criterion recognition and 84.2% for final CVS detection [51]. SurgSmart achieved criterion-specific accuracies ranging from 78.87% to 97.62%; however, complete CVS was present in only 4.33% of the 431 application videos, making overall accuracy particularly sensitive to class prevalence [49].

Several subsequent algorithms were evaluated using related Endoscapes material. LG-CVS achieved an mAP of 63.6 using bounding boxes and 67.3 using segmentation masks [67]. A temporal Swin model achieved an mAP of 67.45% and balanced accuracy of 70.25% at approximately 9 Hz, although end-to-end temporal training did not significantly outperform the comparator [68]. Label-free distillation improved mAP by 3.21 percentage points over the strongest label-free baseline [69], while a Segment Anything-assisted conference method reported an approximately 20% relative improvement in video-level CVS assessment compared with LG-CVS [70]. These studies advanced label efficiency and temporal modeling, but their shared benchmark ancestry prevents their findings from being interpreted as independent clinical replications.

Prospective procedure-level studies moved closer to the intended clinical use. In 40 elective LCs, one YOLOv8 system was reported to detect CVS in every procedure, with complete agreement among three blinded surgeons [52]. A separate network trained using 700 videos was evaluated in real time during 40 elective LCs. It identified 33 of 34 surgeon-reported positive cases and all six surgeon-reported negative cases, corresponding to a reported sensitivity of 97% and specificity of 100% [53]. Both studies were small and predominantly included elective cases. Their reference standards and limited numbers of negative or difficult procedures introduce uncertainty not fully conveyed by the headline performance estimates. At the implementation level, SurgSmart deployment across three hospitals was associated with improved CVS quality scores and improvement in 15 of 18 participating surgeons [50]. This represents the strongest post-deployment process signal in the evidence base, but it was not a comparator-controlled patient-outcome study.

### 3.6. ICG-Assisted and Multimodal Recognition

Three direct studies coupled AI with fluorescence or fluorescence-derived representations. A preliminary real-time report used ICG to support recognition of loose connective tissue and reported a Dice coefficient of 0.60; however, the evaluable denominator was insufficiently clear to support a stable precision estimate [60]. In 113 ICG-guided procedures, a six-class YOLOv7 detector achieved an overall mAP of 0.846, with AP values of 0.864 for the common bile duct and 0.698 for the cystic duct [59]. During short-video testing, common bile duct sensitivity was 99.36%, whereas specificity was only 19.35%, despite an accuracy of 94.39%. The reported metrics imply a test set composition of 468/499 CBD-positive clips (93.79%; 465 true-positive and three false-negative clips) and 31/499 CBD-negative clips (6.21%; six true-negative and 25 false-positive clips). This marked class imbalance explains how high sensitivity and overall accuracy coexisted with poor specificity and illustrates why prevalence, decision thresholds, false-positive burden, and class-specific metrics must accompany overall accuracy.

An ICG-overlay hepatocystic triangle detector reported an AP of 0.859 across 3796 images, although it was unclear whether the training and validation images had been separated at the procedure level [61]. Severe inflammation, subtotal procedures, conversion to open surgery, and other high-difficulty conditions were also excluded or underrepresented in parts of the fluorescence evidence. AI may standardize the interpretation of a complementary visual signal, but the current literature does not establish superiority over fluorescence alone, optimal acquisition timing, or an effect on dissection behavior or patient outcomes.

### 3.7. Robustness in Inflammation, Scarring, Bleeding, and Other Adverse Fields

Only a limited subset of studies directly examined adverse operative conditions. A scar tissue segmentation model developed using 21 acute cholecystitis cases achieved a Dice coefficient of 0.612 and received a mean external expert detection score of 3.98/5 across 20 cases from other centers [62]. A bleeding-robust method used CycleGAN-derived pseudo-nonbleeding images and uncertainty fusion. Dice performance improved significantly compared with a conventional model for all three expert comparisons, with a processing time of 0.06 s/frame; however, only 99 images from 20 patients were evaluated, and a pooled absolute Dice estimate was not available in the indexed report [63].

In the remaining literature, difficult cases were frequently excluded, underrepresented, or insufficiently stratified. Few studies reported prespecified subgroup performance for acute inflammation, severe fibrosis, obesity, active bleeding, smoke, lens contamination, instrument occlusion, distorted traction, anatomical variation, previous intervention, or subtotal and bailout procedures. Robustness across camera systems, hospitals, surgeons, and evolving operative workflows was also rarely quantified. Consequently, the literature is strongest for visible anatomy in selected or retrospectively sampled scenes and weakest for the unstable, ambiguous, and high-risk operative states most likely to benefit from assistance.

### 3.8. Real-Time Feasibility, Human Interaction, and Clinical Applications

Real-time evidence encompassed several materially different modes of implementation. J-SUMMIT studies displayed anatomical landmark overlays prospectively during 10 and 20 procedures [26,54]. The guidance line detector was demonstrated in the operating room [56], and a CVS classifier underwent live procedure-level validation in 40 elective cases [53]. ICG-assisted connective tissue recognition was reported as a small live demonstration [60]. A separate real-time support model provided feedback concerning clipper-tip visibility, demonstrating an adjacent safety application rather than direct anatomical certification [78]. By contrast, SurgFlow processed three cases in shadow mode: the system received a secondary video stream and generated predictions that were not displayed to the operating surgeon [28]. Offline video experiments evaluated changes in anatomical interpretation or incision selection [58,65], whereas SurgSmart examined post-deployment CVS quality scores over several months [50]. Conflating these settings would materially overstate clinical maturity.

The prospective studies established the feasibility of real-time processing and operating-room integration but involved small and generally selected samples. Latency, downtime, recovery from occlusion, alert frequency, surgeon override, and failure-management procedures were inconsistently reported. No study randomized patients or operating teams to AI-assisted versus unassisted laparoscopic cholecystectomy using prespecified clinical outcomes.

Companion evidence broadened the range of plausible applications without closing this effectiveness gap. In a randomized coaching study involving 18 surgeons from 10 hospitals, AI-supported coaching improved the laparoscopic cholecystectomy rating-form score and increased CVS achievement [30]. An educational study demonstrated better anatomical segmentation performance among students using AI-supported learning than among self-directed learners [31]. Automated video review also outperformed operative notes for retrospective documentation of CVS in a 279-video series, although discrimination remained moderate, with an area under the receiver-operating-characteristic curve of 0.77 [77]. These findings support potential near-term roles in video triage, quality assurance, structured documentation, coaching, and education.

### 3.9. Methodological Appraisal and Clinical Readiness

Application of the review-specific readiness framework placed five of the 39 direct reports at Stage 0, 19 at Stage 1, eight at Stage 2, six at Stage 3, and one at Stage 4 (Table 3). Thus, 24 of 39 reports remained at the offline proof of concept or internal validation level. The six Stage 3 studies demonstrated prospective operating-room feasibility, live validation, or shadow-mode integration, but none provided comparator-controlled effectiveness evidence [26,28,53,54,56,60]. The sole Stage 4 assignment reflected post-deployment process surveillance rather than patient-level outcomes [50]. The assigned stage represented the highest translational step demonstrated by each report and did not indicate regulatory status, product approval, overall methodological quality, or a low risk of bias.

Recurring methodological limitations included nonrepresentative or incompletely described case mix; limited external, temporal, and multicenter validation; uncertain procedure-level separation in some image-based dataset splits; heterogeneous expert annotations without adjudicated consensus; class imbalance; reliance on aggregate metrics; and limited reporting of calibration, uncertainty, threshold selection, missing frames, and failure cases. In the public benchmark literature, repeated reuse of Endoscapes and Cholec80 enabled reproducible technical comparison but narrowed the diversity of independent clinical evidence. In private datasets, limited access to annotations, code, and complete deployment specifications impeded external replication.

The available evidence supports technical feasibility and selected intermediate clinical functions, particularly automated video localization, documentation support, quality review, training, and perceptual prompting. It does not support autonomous anatomical certification or authorization to dissect or divide structures. The decisive question is whether prospectively governed systems improve surgeon performance across institutions, devices, and difficult anatomical conditions without introducing false reassurance, alert fatigue, or automation bias.

## 4. Discussion

### 4.1. Principal Findings and Added Value of This Review

Collectively, the available literature shows that computer vision can identify selected anatomical landmarks, segment visible hepatocystic structures, assess elements of the critical view of safety, generate spatial safety prompts, and operate prospectively on intraoperative video. However, the readiness distribution summarized in Section 3.9 remains concentrated at the proof of concept and internal validation stages, and the only Stage 4 report evaluated a surgical-process measure rather than bile duct injury or morbidity [26,28,32,33,34,35,50,53,54,56,60].

Four interpretive boundaries are central. First, recognition of what is visible in the image is not equivalent to knowledge of the complete three-dimensional biliary anatomy, particularly when a duct is occult, compressed, inflamed, or outside the field of view. Second, frame-level overlap or classification performance is not equivalent to improved surgeon perception, and improved perception is not equivalent to safer operative action. Third, documentation of the CVS and achievement of the CVS are different endpoints. Fourth, the publication count exceeds the number of independent data-generating programs because several reports arose from successive development within the Oita/J-SUMMIT, CAMMA/EndoDigest/Endoscapes, GoNoGoNet, and SurgSmart families. These linked studies legitimately answer different questions, but their results cannot be interpreted as independent replications [18,19,21,22,23,24,25,26,28,48,49,50,54,62,63,65,66,67,68,69,70].

The principal contribution of the present review is therefore not a rank ordering of algorithms. It is a clinically anchored interpretation of what each output means at a specific decision point in laparoscopic cholecystectomy (LC), how closely the validation resembles the intended use, and what evidence remains necessary before the output can influence tissue division. This distinction is important because a model with modest segmentation metrics may still be useful for postoperative video triage, whereas a visually impressive overlay may be unsuitable for live dissection guidance if its false-negative behavior, calibration, latency, or response to adverse operative fields is unknown [37,38,41,79].

Previous reviews have addressed complementary but distinct aspects of artificial intelligence in laparoscopic cholecystectomy. The 2024 narrative review surveyed broad applications encompassing operative phase recognition, anatomy, and cholecystitis [39], while the 2025 systematic reviews concentrated primarily on automated anatomical structure and landmark recognition [32,33]. A subsequent review focused on AI-assisted safety assessment [34], and the most comprehensive computer vision review included studies published through March 2026 across the broader spectrum of laparoscopic cholecystectomy applications [35]. The present review extends this literature through 15 August 2026 and focuses specifically on AI-assisted recognition of hepatocystic anatomy and its relationship to safe operative decision-making. Its additional contributions include detailed characterization of 39 direct model development or evaluation reports, explicit mapping of overlapping datasets and research families, preservation of the original unit and aggregation level of performance metrics, application of a staged clinical readiness framework, and dedicated evaluation of difficult cholecystectomy, multimodal imaging, human–AI interaction, and implementation requirements. Thus, its added value lies not in duplicating previous performance summaries but in translating heterogeneous technical evidence into clinically interpretable readiness and deployment recommendations.

### 4.2. From Visual Recognition to Safe Operative Decision Support

Anatomical recognition during LC is a dynamic inference problem rather than a static labeling exercise. Traction changes tissue relationships; dissection progressively reveals structures; the camera moves; and blood, smoke, fat, instruments, clips, and the gallbladder itself intermittently obscure the operative field. The surgeon integrates this evolving visual evidence with tactile feedback, preoperative information, the expected course of the operation, and an internal model of possible anatomical variants. Most current AI systems observe only the two-dimensional video stream and should therefore be understood as bounded perceptual aids, not as complete representations of operative anatomy [3,5,6,7,37,80].

The reviewed tasks occupy different levels of clinical inference. Landmark detectors answer whether a recognizable visual feature is present and where it is located [18,55,56]. Segmentation models assign pixels to visible structures or regions [19,20,74]. CVS systems estimate whether one or more predefined visual criteria are present or identify a video interval suitable for assessment [21,22,51,67]. Go/No-Go and alert-zone models encode expert-defined spatial conventions intended to influence attention or dissection behavior [19,24,58]. None of these outputs independently establishes that an unvisualized duct is absent, that a proposed plane is avascular, or that clipping and division are safe.

Error consequences are also asymmetric. Failure to detect a small cystic duct or common bile duct may be clinically more important than modest boundary imprecision around the liver or gallbladder. Conversely, overinclusive hazard zones or frequent false-positive duct alerts may obscure the image, interrupt workflow, and erode trust. The prospective decline in Go-zone sensitivity compared with internal testing, the low absolute performance for several small structures in relation-aware detection, and the low specificity reported for common bile duct detection in an indocyanine green (ICG)-dominant video set show why aggregate accuracy or mean overlap cannot define safety [24,59,72]. Clinically meaningful evaluation must specify which error is being measured, when it occurs, and what action it could provoke or suppress [38].

Accordingly, anatomy-aware AI should not present a positive prediction as permission to dissect or divide tissue. A safer interaction model is to provide confidence-bearing information, suppress output when image quality or model certainty is inadequate, and frame alerts as prompts to reassess exposure, traction, the CVS, or the need for a bailout strategy. The operative decision must remain with the surgeon and remain governed by established safe cholecystectomy principles. An absent or uncertain AI output should default to conventional surgical judgment rather than be interpreted as evidence that the field is safe [3,4,6,7,10].

### 4.3. Clinical Applications with the Strongest Near-Term Rationale

The most credible near-term applications are those that reduce cognitive or administrative burden without converting an imperfect prediction into an irreversible operative action. Automated localization of cystic duct division and extraction of a candidate CVS video segment can substantially reduce the time required for postoperative review, although multicenter results show that the selected clip still requires human confirmation [21,48]. Similarly, automated assessment can supplement operative notes with objective video-based evidence, facilitate structured archiving, and identify cases requiring expert review [77]. These uses extend existing recommendations for visual CVS documentation rather than replacing them [11,12,81].

Education, coaching, and quality improvement represent a second pragmatic domain. AI-supported coaching improved procedure-specific performance and CVS achievement in a randomized study of practicing surgeons, while an educational study reported improved anatomical segmentation among learners [30,31]. SurgSmart studies further suggest that scalable video review can characterize CVS quality and support longitudinal improvement at the surgeon or institutional level [49,50]. Such programs should be developmental and transparent. Because model labels and case mix remain imperfect, automated scores should not be used in isolation for credentialing, punitive surveillance, or professional evaluation.

Real-time overlays may be valuable as selective attention prompts. Prospective studies have shown that landmark, guidance line, and CVS outputs can be generated during live operations, and offline experiments indicate that overlays can change anatomical interpretation or safe-incision selection [26,53,54,56,58,65]. Nevertheless, these reports involved small samples, selected procedures, or simulated decision settings. The appropriate current claim is feasibility and potential cognitive assistance, not prevention of BDI. Silent or shadow-mode deployment, as used by SurgFlow, is particularly useful before surgeon-facing activation because it permits measurement of latency, uptime, discordant predictions, and domain shift without exposing the operator to unvalidated advice [28,40].

**Figure 2:**
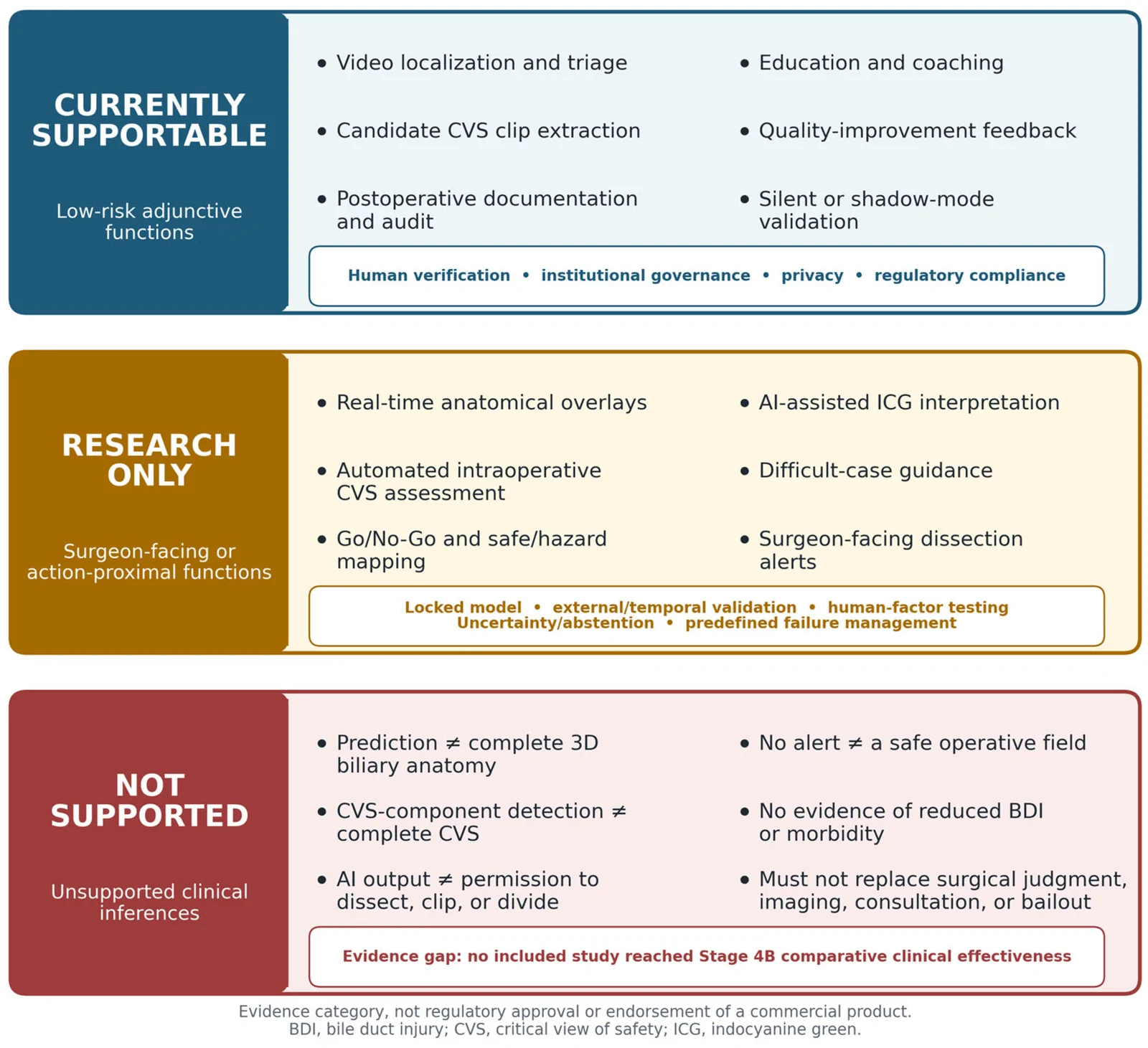
Current clinical recommendations for anatomy-aware AI during laparoscopic cholecystectomy. Note: These recommendations describe the level of use supported by the current evidence and do not constitute regulatory approval or endorsement of a specific commercial system. No included study reached Stage 4B comparative clinical effectiveness evidence.

The clinical acceptability of an AI application should be proportional to its proximity to an irreversible operative action and to the potential consequences of model error. Based on the available evidence, current adjunctive applications, research-only functions, and unsupported clinical inferences are summarized in Figure 2 [37,45,46].

### 4.4. Generalizability, Reference Standards, and Metric Interpretation

Generalizability remains the major technical barrier. The contrast between very high internal segmentation performance and poor qualitative transfer to private images in one study is a direct demonstration of domain shift [64]. More broadly, external performance declined for Go-zone mapping and multicenter CVS-clip extraction compared with development-center evaluation [24,48]. Variation in camera systems, compression, illumination, operative technique, institutional case mix, and annotation practice can alter the image distribution even before differences in inflammation or anatomy are considered. Internal random frame splits cannot characterize this problem and may produce optimistic estimates when visually adjacent frames from the same procedure occur in both development and test datasets [38,43,44].

Procedure-level separation should therefore be the minimum standard for model development, with patient-, surgeon-, center-, and time-level independence reported when applicable. External validation should preserve the prespecified model and decision threshold, include all consecutive eligible procedures, and report failures of video acquisition or inference. Performance should be stratified by center, device, surgeon experience, operative difficulty, and clinically important subgroups rather than summarized only by a pooled mean. Temporal validation is also necessary because image pipelines, operative equipment, and local practice may change after model development [38,43,44].

The reference standard is equally consequential. CVS assessment is not perfectly reproducible among surgeons, particularly for complete clearance of the hepatocystic triangle, and difficult inflammation reduces the likelihood that the CVS can be achieved [8,14,15,82]. Labels created by a single reviewer or by consensus without reporting disagreement can conceal uncertainty rather than eliminate it. Future studies should use an explicit annotation manual, multiple appropriately experienced raters, blinded independent assessment, adjudication of discordance, and quantitative inter-rater agreement. When uncertainty is genuine, probabilistic or ordinal labels may represent the clinical construct more faithfully than an artificial binary ground truth.

Metric selection should reflect intended use and error cost. Small-structure detection requires class-specific sensitivity, precision, false detections per unit time, and temporal persistence; segmentation requires class-wise overlap and boundary measures; procedure-level CVS assessment requires sensitivity, specificity, predictive values, calibration, and confidence intervals; and real-time systems require latency, dropped-frame behavior, uptime, and recovery after occlusion. Calibration and decision-curve analysis are particularly relevant when an output triggers attention or escalation. Qualitative surgeon approval, overall accuracy, or a single mean average precision value cannot substitute for a complete error profile [38,44].

Open resources such as Cholec80, Cholec80-CVS, CholecSeg8k, and Endoscapes have accelerated reproducibility and standardized comparison [83,84,85,86]. Their repeated use, however, does not create independent clinical validation. Benchmark governance should maintain hidden procedure-level test sets, document dataset lineage, prevent repeated tuning to the test set, and include challenging and geographically diverse cases. The emerging Society of American Gastrointestinal and Endoscopic Surgeons (SAGES) CVS Challenge may support such harmonization, but preprint benchmark findings should remain provisional until peer review and independent replication [87].

### 4.5. Difficult Cholecystectomy and Multimodal Imaging

The clinical value proposition of anatomy-aware AI may be greatest during difficult cholecystectomy, yet the supporting evidence is least secure in this setting. Acute inflammation, dense fibrosis, a contracted gallbladder, impacted stones, active bleeding, obesity, anatomical variation, previous intervention, and subtotal or other bailout procedures were frequently excluded, underrepresented, or insufficiently characterized. The term “difficult cholecystectomy” should therefore not be used as an unqualified binary descriptor. Future studies should adopt a standardized minimum reporting dataset that distinguishes acute from chronic cholecystitis; reports body mass index and previous endoscopic retrograde cholangiopancreatography, cholecystostomy, or upper-abdominal surgery; grades inflammation and operative difficulty using a validated system such as the Parkland or Nassar classification; and documents dense adhesions, fibrosis, anatomical variation, and active bleeding [82,88,89,90,91]. Operative outcomes should include achievement or non-achievement of the critical view of safety, subtotal cholecystectomy or another bailout procedure, conversion to open surgery and its indication, and intraoperative bleeding. When subtotal cholecystectomy is performed, the fenestrating or reconstituting technique should be specified. AI performance should be stratified according to these characteristics using procedure-level denominators and uncertainty estimates rather than being reported only as a pooled average.

Initial work on scar tissue segmentation and bleeding-robust image translation is clinically relevant because it addresses adverse visual conditions rather than removing them from the dataset [62,63]. However, these studies were small and did not establish reliable performance across the full spectrum of acute cholecystitis. Robustness testing should include blood, smoke, condensation, lens contamination, motion blur, instrument occlusion, extreme traction, and sudden camera changes, together with explicit image quality or out-of-distribution detection. The system should be able to abstain when the input is unsuitable.

ICG fluorescence offers a complementary signal for extrahepatic biliary visualization and has an established non-AI clinical literature [92,93,94,95]. The reviewed AI–ICG studies demonstrate technical feasibility for detecting ducts, connective tissue, or the hepatocystic triangle [59,60,61], but they do not establish superiority over fluorescence interpretation alone. Fluorescence is affected by dose, timing, tissue thickness, inflammation, equipment, and background signal; an algorithm may amplify these limitations as well as the useful signal. Comparative studies should therefore evaluate white light, ICG alone, AI applied to white light, and AI-assisted ICG within the same clinical pathway, while reporting whether multimodal output changes dissection behavior or merely improves detection metrics.

### 4.6. Human Factors and Responsible Interface Design

Intraoperative AI is a team intervention, not only a model. Its effect depends on when information appears, how it is displayed, how often it is wrong, whether the operator understands its limitations, and how it interacts with workload and established safety routines. A continuously colored operative field may create visual clutter, whereas an intermittent prompt delivered at a decision point may be easier to interpret. Conversely, an alert that appears only after a hazardous maneuver has begun may be technically accurate but clinically ineffective. Human factor evaluation must therefore be designed alongside model development rather than added after accuracy has been optimized [37,40,41,80].

The principal behavioral risks are automation bias, false reassurance, alert fatigue, attentional tunneling, and deskilling. A confident but incorrect overlay may anchor the surgeon to an erroneous anatomical model; repeated low-value alerts may be ignored; and a junior operator may overestimate the authority of a visually sophisticated display. Interface design should communicate uncertainty without demanding complex interpretation, distinguish confirmed visible structures from inferred regions, and avoid green-light language that implies authorization to divide tissue. The ability to dismiss or hide an overlay, request a clean video view, and document disagreement should be preserved [37,41,80].

Existing human-interaction evidence is encouraging but preliminary. Anatomy overlays changed surgeon responses in an offline experiment, an alert-zone pilot improved video-based safe-incision selection, and survey-based work has begun to examine surgeon perceptions and visual-attention guidance [58,65,96]. The CVS Copilot 3.0 preprint further illustrates active investigation of the human–machine interface, but its non-peer-reviewed status limits its evidentiary weight [97]. Future live studies should measure task completion, decision accuracy, response time, visual attention, mental workload, trust calibration, override behavior, near misses, and recovery from deliberately sampled model failures.

Responsibility must remain explicit. AI output should supplement, not replace, the surgical time-out, CVS confirmation, intraoperative imaging when indicated, consultation with an experienced colleague, or bailout surgery. Institutions should define who monitors the system, how technical failure is communicated, how discordance is documented, and how adverse events potentially related to AI are reviewed. Training should include false-positive and false-negative examples so that users learn when not to rely on the system [3,4,98].

### 4.7. Clinical Validation, Regulation, and Implementation

**Figure 3:**
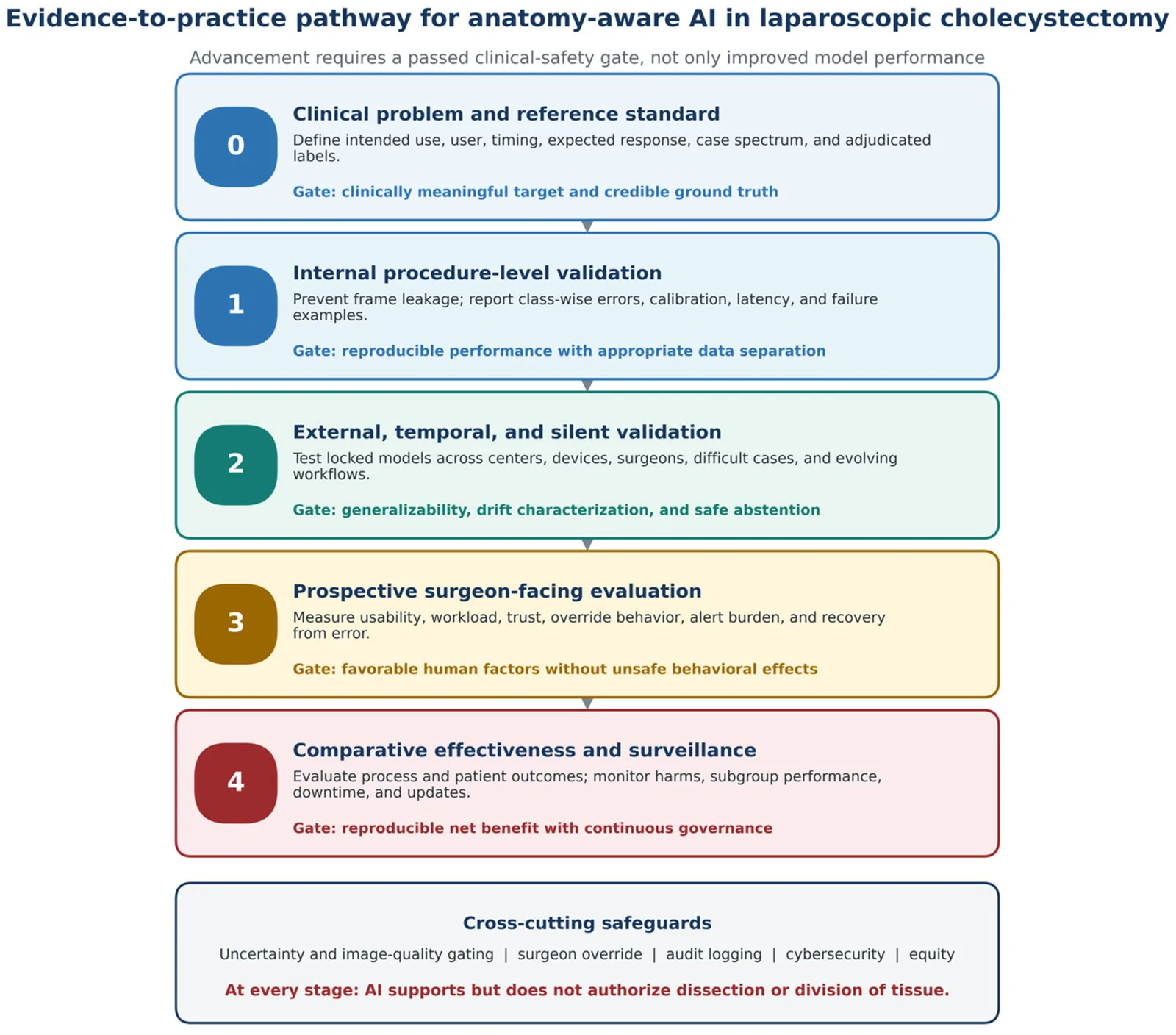
Proposed staged pathway for the responsible translation of anatomy-aware AI in LC. The stages mirror the review-specific clinical readiness framework. Advancement requires both technical validity and passage through a clinical-safety gate; technical performance alone does not authorize dissection or tissue division. The framework is informed by contemporary guidance concerning AI reporting, clinical validation, medical device evaluation, ethics, and regulation [38,40,43,44,45,46,98,99,100,102].

Translation should follow a staged pathway in which the strength of evidence increases with the proximity of the AI output to an irreversible surgical action (Figure 3). Early development should establish a clinically justified intended use, representative reference standard, and procedure-level data independence. This should be followed by locked-model external and temporal validation, prospective silent deployment, and then small live studies focused on usability and failure behavior. Comparative evaluation and post-deployment surveillance should occur only after these gates are passed [38,40,43,44,45,46].

Clinical evaluation should use prespecified algorithmic, human factor, process, and patient endpoints. These levels are complementary but non-interchangeable: gains in technical performance or workflow may justify further evaluation but cannot be assumed to reduce bile duct injury or morbidity. Because major bile duct injury is rare, definitive benefit assessment will likely require multicenter comparative designs and prolonged surveillance rather than substitution of technical accuracy for patient safety [1,2,38,40].

Pragmatic multicenter designs may combine cluster randomization or stepped-wedge implementation with blinded video adjudication and registry linkage. Prespecified intermediate outcomes can provide sensitivity to change, while large longitudinal cohorts and post-market surveillance accumulate sufficient exposure to identify uncommon injuries or new failure patterns. Analyses should include learning curves, surgeon experience, urgent versus elective surgery, operative difficulty, center effects, device type, and differential benefit or harm across patient groups. Trial protocols and reports should follow SPIRIT-AI and CONSORT-AI, while early live evaluations should follow DECIDE-AI [40,99,100,101].

Regulatory and governance requirements depend on the system’s intended use, degree of influence on clinical decisions, and integration with a medical device. In the European context, some anatomy recognition systems may enter overlapping high-risk-AI and medical device pathways, requiring quality management, risk control, technical documentation, human oversight, performance monitoring, and management of software updates [102]. Regardless of jurisdiction, governance should address data provenance, privacy, cybersecurity, interoperability, version control, bias, incident reporting, and responsibility for maintenance. Ethical deployment also requires transparency regarding limitations and equitable evaluation across hospitals with different resources and patient populations [98,102].

Post-deployment monitoring is essential because performance can drift without an obvious software malfunction. Changes in camera hardware, compression, surgical technique, clinical case mix, or local workflow may alter inputs; user behavior may also change in response to the tool. Monitoring should therefore include input-quality statistics, confidence distributions, alert frequency, discordance and override rates, technical downtime, subgroup performance, operative-process measures, and adverse events. Model updates should trigger documented revalidation proportional to the change rather than being introduced as invisible background improvements [37,45,79,98].

### 4.8. Future Research Priorities

Future systems should move beyond isolated-frame recognition toward temporally coherent scene understanding. Anatomical relationships evolve through traction and dissection, and a reliable model should use prior frames to distinguish transient occlusion from true disappearance, detect when the visual field is inadequate, and recognize the operative step in which a prediction is relevant. Relational and graph-based approaches, temporal transformers, and multitask models already point in this direction [67,68,75,103]. Their value should be assessed through improved calibration and failure handling, not only incremental gains on a reused benchmark.

Foundation segmentation and vision–language methods may reduce annotation burden and support structured explanations, but they introduce additional risks. A fluent description can be anatomically incorrect, and a broadly pretrained model may inherit undocumented biases or fail under laparoscopic domain shift. Segment Anything-assisted CVS assessment and emerging structured reasoning models should therefore be evaluated using locked prompts, predefined output ontologies, external testing, hallucination analysis, and explicit abstention [70,104]. Likewise, tissue affordance prediction could eventually connect anatomy to tool- and action-specific risk, but current work remains preclinical and should not be represented as evidence for clinical dissection guidance [105].

Progress also depends on data infrastructure. Multicenter video consortia should retain procedure-level metadata, standardized difficulty grading, device information, operative phases, adverse visual conditions, bailout strategies, and longitudinal outcomes while protecting privacy. Federated or privacy-preserving development may broaden representation when video sharing is restricted, but local heterogeneity must remain visible in the analysis. Public datasets and challenges should publish lineage and overlap maps so that apparent external validations can be distinguished from repeated testing on related material [79,83,85,86,87].

The highest-priority research question is not which architecture achieves the best single metric, but which clearly defined clinical function produces a reproducible net benefit. Studies should prespecify the intended users, timing, display, decision threshold, expected response, contraindications, and failure-management plan. A core outcome framework for anatomy-aware LC systems would improve comparison by requiring class-specific technical metrics, calibration, image quality failure, latency, surgeon response, CVS documentation, bailout behavior, near misses, BDI and bile leak, other morbidity, and unintended consequences. Independent replication and prospective multicenter evaluation should be rewarded more strongly than repeated optimization on established benchmarks [35,36,38,40,44].

### 4.9. Strengths and Limitations of This Review

This review used a structured and reproducible search of five bibliographic databases through 15 August 2026, supplemented by citation tracking and targeted searches. It included 105 verified references, distinguished 50 primary AI reports from 55 contextual or methodological sources, and characterized 39 direct model development or evaluation reports in detail. The synthesis preserved the original unit and aggregation level of reported metrics and explicitly mapped overlapping datasets and research families. The clinically oriented task taxonomy and readiness framework represent the principal interpretive contributions of this review.

Several limitations should be acknowledged. This was a narrative review rather than a registered systematic review, and no formal meta-analysis was performed. No formal design-specific risk of bias instrument was applied because the included evidence encompassed heterogeneous technical, observational, diagnostic, human factor, and early clinical-evaluation designs. Although the structured domain-based appraisal improved methodological transparency, it is not equivalent to a validated study-level risk of bias assessment, and the clinical readiness classification remains partly judgment-based. Residual selection, reference standard, data leakage, and selective-reporting biases may therefore affect the reported performance estimates. Substantial heterogeneity in anatomical targets, denominators, metrics, and validation designs precluded quantitative pooling. Some conference reports, preprints, and emerging studies provided incomplete methodological information and may change following peer review. English-language full-text availability may have limited inclusion despite the absence of a database language restriction. Publication bias, unavailable code or data, and dependence among research groups may further overstate reproducibility. Finally, the absence of demonstrated patient-level benefit reflects the limitations of the available evidence and should not be interpreted as evidence that such benefit is impossible.

## 5. Conclusions

Artificial intelligence can recognize selected hepatocystic landmarks, segment visible anatomy, assess components of the critical view of safety, generate spatial prompts, and support intraoperative or postoperative video analysis during laparoscopic cholecystectomy. The strongest near-term applications are automated documentation and video triage, education and coaching, quality assurance, and carefully governed perceptual prompting. However, most studies remain retrospective or internally validated, difficult operative conditions are underrepresented, and no comparator-controlled evidence currently demonstrates a reduction in bile duct injury or other patient-level morbidity.

Clinical implementation should therefore remain adjunctive, staged, and proportionate to the consequence of model error. Progress toward routine deployment requires procedure-level data independence, multicenter and temporal validation, explicit uncertainty and abstention mechanisms, prospective human factor evaluation, comparative-effectiveness studies, and continuous post-deployment surveillance.

## Figures and Tables

**Table 1:** Current evidence summary and clinical deployment recommendations for AI-assisted recognition of hepatocystic anatomy.

AI Function	Evidence Stage	Strongest Supporting Evidence	Current Potential Use	Principal Failure Risk	Clinical Recommendation
CVS recognition and automated documentation	Stages 1–4A	Internal and multicenter validation, prospective feasibility, and post-deployment process surveillance [21,48,49,50,51,52,53]	Video localization, documentation, retrospective audit, training, and quality improvement feedback	Variable CVS prevalence, imperfect reference standards, and absence of patient-level effectiveness endpoints	May be used as an adjunct for documentation or quality improvement under institutional governance; it should not certify completion of the CVS
Anatomical landmark detection and segmentation	Stages 0–3	Multiple internally validated models and selected small prospective intraoperative demonstrations [18,23,26,54,55,56]	Educational overlays, perceptual prompting, and anatomical orientation	Domain shift, small-structure detection failure, obscuration by blood, smoke, fibrosis, or adipose tissue	Research or closely supervised feasibility use only
Safe zone, hazardous zone, and Go/No-Go segmentation	Stages 1–2	Internal validation, independent external validation, and simulated human factor evaluation [19,24,25,57,58]	Training, retrospective analysis, and investigational dissection guidance	Inconsistent spatial definitions, reduced external sensitivity, and false reassurance	Research-only; it must not authorize dissection or division of structures
ICG-assisted biliary anatomy recognition	Stages 1–3	Internal image/video validation and preliminary live intraoperative feasibility [59,60,61]	Adjunctive fluorescence interpretation and anatomical prompting	Prevalence-dependent performance, low specificity for selected structures, and limited difficult-case validation	Research-only pending prospective multicenter validation
Recognition under difficult operative conditions	Stages 1–2	External evaluation in acute cholecystitis and limited bleeding-robustness experiments [62,63,64]	Investigational support in inflammation, scar tissue, or bleeding	Small cohorts, heterogeneous difficult-case definitions, and substantial domain shift	Insufficient evidence for routine clinical deployment
Human factor and intraoperative decision support	Stages 1–3	Paired offline behavioral studies, shadow-mode feasibility, and small live demonstrations [28,58,65]	Training, perceptual prompting, and workflow evaluation	Automation bias, alert fatigue, distraction, and inappropriate reliance	Use only within prospective, human factor-governed research protocols
Patient-level clinical effectiveness	Stage 4B not reached	No comparator-controlled evidence demonstrating reduced BDI, conversion, morbidity, or mortality	None established	Incorrect extrapolation from technical accuracy to clinical benefit	No current evidence supports autonomous anatomical certification, “safe-to-divide” authorization, or inference of improved patient outcomes

Stage 4A denotes post-deployment surveillance of technical reliability, human interaction, or operative-process outcomes without comparator-controlled patient-level effectiveness. Stage 4B denotes comparative clinical-effectiveness evidence demonstrating patient-level safety or benefit. No included study reached Stage 4B. Detailed study-level characteristics and performance estimates are provided in Supplementary Table S1. Abbreviations: BDI, bile duct injury; CVS, critical view of safety; ICG, indocyanine green.

**Table 2:** Clinical taxonomy of AI tasks for hepatocystic anatomy recognition and safety support during laparoscopic cholecystectomy.

Task Group	Clinical Question	AI Output/Unit	Typical Metrics	Representative Evidence	Principal Interpretive Caveat
**1. Landmark/anatomical structure recognition**	Which visible safety-critical structures or landmarks are present, and where are they located?	Bounding boxes, points, labels, or guidance lines at the frame or image level	AP/mAP, precision, recall, sensitivity, specificity, detection rate, and latency	[18,23,55,56,59,66,71,72,73]	Small or occluded ducts remain difficult to detect; a visible landmark does not represent a complete map of the biliary anatomy.
**2. Pixel-level semantic or instance segmentation**	Which pixels belong to the gallbladder, liver, ducts, hepatocystic triangle, connective tissue, scar tissue, or another target?	Per-pixel mask or multiple masks, sometimes incorporating temporal propagation	IoU, Dice/F1 score, class-wise sensitivity, PPV, boundary error, and frame rate	[19,20,22,57,58,62,63,64,74,75,76]	High aggregate overlap may conceal failure involving small structures; random frame-level data partitioning can inflate apparent generalizability.
**3. CVS localization, component recognition, or achievement assessment**	When is the CVS assessable, are its three criteria present, and has the complete CVS been achieved?	Time point or video clip, criterion-level probability, and frame-, video-, or procedure-level classification	Temporal localization error, mAP, balanced accuracy, sensitivity, specificity, and inter-rater agreement	[21,22,48,49,50,51,52,53,67,68,69,70]	Documentation, component recognition, and complete CVS achievement are distinct endpoints; low prevalence and reference-label ambiguity materially influence performance estimates.
**4. Safe/hazard zone mapping and dissection guidance**	Where should dissection or energy application be encouraged, discouraged, or paused?	Go/No-Go, safe/hazard, or alert-zone mask and incision-site prompt	IoU, Dice/F1 score, sensitivity, specificity, PPV, and change in surgeon response	[19,24,25,57,58,65]	Spatial zones encode expert-defined conventions rather than an invisible anatomical truth; false reassurance and automation bias remain important safety concerns.
**5. Multimodal or ICG-assisted recognition**	Can fluorescence or another complementary representation improve visualization of ducts or dissection planes?	Anatomical bounding boxes or masks derived from white-light imaging combined with fluorescence or synthetic paired images	AP/mAP, sensitivity, specificity, F1/Dice score, and latency	[59,60,61,63]	Standardized acquisition and fluorescence timing are required; performance may be prevalence-dependent and does not establish superiority over fluorescence interpretation alone.
**6. Real-time deployment, documentation, education, and human factors**	Can the system operate reliably within the surgical workflow and improve anatomical recognition, documentation, learning, or operative behavior?	Live overlay, shadow-mode prediction, automated video clip or report, coaching, and quality feedback	System availability, latency, technical failure rate, usability, decision change, process or quality score, and patient outcomes	[26,28,30,31,47,50,52,53,54,56,58,60,65,77,78]	Technical feasibility and improvements in operative processes precede—and cannot substitute for—comparative-effectiveness evidence and demonstrated patient-level safety benefits.

Abbreviations: AI, artificial intelligence; AP, average precision; CVS, critical view of safety; F1, harmonic mean of precision and recall; ICG, indocyanine green; IoU, intersection-over-union; mAP, mean average precision; PPV, positive predictive value. Task groups are not mutually exclusive.

**Table 3:** Structured domain-based methodological appraisal of the included evidence. Panel (**A**): Cross-cutting methodological and translational appraisal. Panel (**B**): Highest clinical readiness stage demonstrated by each direct report (*n* = 39).

(**A**)				
**Appraisal Domain**	**Synthesis of the Evidence**			**Implication for Interpretation**
**Clinical representativeness**	Most datasets were retrospective and selectively sampled. Elective or visually interpretable cases predominated; severe inflammation, fibrosis, bleeding, variant anatomy, bailout procedures, and conversions were frequently excluded, uncommon, or not analyzed separately.			Reported performance may not generalize to the high-risk operative fields in which assistance is most needed.
**Dataset independence and reuse**	Several reports originated from the linked Oita/J-SUMMIT, CAMMA/EndoDigest/Endoscapes, GoNoGoNet, or SurgSmart research programs. Public benchmarks improved comparability but generated multiple publications using related datasets.			The number of publications must not be interpreted as the number of independent replications.
**Data partitioning and leakage control**	Procedure- or video-level partitioning was explicit in several mature datasets, but some image- or frame-based studies employed random splits or did not clearly exclude dependence involving frames from the same procedure [61,64,74].			Highly correlated frames may inflate internal performance estimates and produce artificially narrow confidence intervals.
**Reference standard**	Reference labels ranged from assessments by individual surgeons to expert committees. Consensus procedures, adjudication, and inter-rater reliability were reported inconsistently. Some live studies used surgeon-reported CVS achievement as the comparator [53].			The apparent model ceiling and estimated error rate depend on an uncertain and sometimes subjective ground truth.
**Metrics and class imbalance**	IoU, Dice/F1, AP/mAP, accuracy, balanced accuracy, and temporal localization error were reported inconsistently. High aggregate accuracy occasionally coexisted with low class-specific sensitivity or specificity [24,59].			Class-specific, threshold-dependent, and clinically weighted errors are more informative than a single headline performance value.
**External, temporal, and multicenter validation**	Independent validation remained uncommon. Where such validation was performed, performance occasionally declined [24,48], or qualitative transfer failed despite nearly perfect internal metrics [64].			Generalizability cannot be inferred from internal cross-validation, even when development data originate from geographically diverse institutions.
**Robustness and failure analysis**	Only a few studies specifically investigated scar tissue or bleeding [62,63]. Smoke, lens contamination, instrument occlusion, tissue deformation, device changes, atypical anatomy, and missing frames were rarely evaluated systematically.			Failure modes and safe-degradation behavior remain insufficiently characterized.
**Calibration and uncertainty**	Probability calibration, uncertainty thresholds, abstention mechanisms, alarm burden, and prospective threshold locking were rarely reported. Uncertainty fusion in reference [63] represented a robustness method rather than evidence of calibrated clinical confidence.			A visually confident overlay may remain poorly calibrated and may create false reassurance.
**Real-time integration and human factors**	Live display, real-time validation, shadow-mode deployment, offline simulation, and post-deployment monitoring were represented but were frequently conflated. Latency, downtime, surgeon override, alert fatigue, attentional effects, and automation bias were incompletely assessed.			Operating-room feasibility is necessary but does not establish safe or beneficial human–AI interaction.
**Clinical effectiveness and safety**	No direct report evaluated reduction in BDI or another patient-level endpoint. Reference [25] represented a retrospective association, whereas reference [50] demonstrated improvement in a post-deployment CVS-process measure only.			The available literature cannot support claims of reduced BDI, morbidity, conversion, reintervention, or healthcare utilization.
**Transparency and reproducibility**	Data, annotations, source code, model versions, preprocessing procedures, hardware specifications, and funding or conflict-of-interest disclosures were inconsistently available. Conference reports contained limited extractable methodological detail.			Independent replication, technical auditing, and assessment of model change control remain difficult.
(**B**)				
**Stage**	**Definition**	***n***	**References**	**Evidence Interpretation**
**Stage 0**	Offline proof of concept	5	[61,66,73,74,76]	Technical concept or internal experiment without a clearly independent clinical test cohort.
**Stage 1**	Internal held-out or cross-validated evaluation	19	[18,19,20,21,22,23,51,55,57,59,63,65,67,68,69,70,71,72,75]	Largest evidence stratum; supports model development but does not establish transportability or clinical effectiveness.
**Stage 2**	Temporal, external, or multicenter validation	8	[24,25,48,49,52,58,62,64]	Includes external transfer, multicenter analysis, or simulated human factor evidence; methodological strengths and observed failure modes varied.
**Stage 3**	Prospective real-time feasibility or operating-room human factor evaluation	6	[26,28,53,54,56,60]	Demonstrated live or shadow-mode feasibility in small cohorts; no study provided a comparator-controlled patient-level outcome.
**Stage 4A** **.**	Post-deployment surveillance of technical, human factor, or operative-process outcomes	1	[50]	Improvement in a CVS-process measure but did not evaluate comparator-controlled patient outcomes.
**Stage 4B**	Comparative clinical effectiveness demonstrating patient-level safety or benefit	0		No included report demonstrated a reduction in BDI, morbidity, or another patient-level outcome.

*Table 3 notes:* The clinical readiness framework represents an interpretive synthesis developed for this review and informed by staged clinical AI and surgical innovation evaluation frameworks [40,43,44,45,46]. It is neither a validated numerical quality score nor a regulatory classification. The assigned stage indicates the highest translational step demonstrated by each report, not model accuracy, product approval, overall methodological quality, risk of bias, or suitability for autonomous use. Counts sum to 39 direct model development or evaluation reports. Abbreviations: AI, artificial intelligence; AP, average precision; BDI, bile duct injury; CVS, critical view of safety; F1, harmonic mean of precision and recall; IoU, intersection-over-union; mAP, mean average precision.

## Data Availability

No new datasets were generated or analyzed in this study. Data supporting the evidence synthesis are available from the corresponding author upon reasonable request, catalin.cosma@umfst.ro.

## Supplementary Materials

The following supporting information can be downloaded at https://www.mdpi.com/article/10.3390/medicina62091705/s1. Table S1: Characteristics, validation design, principal performance, and translational status of 39 direct model-development or evaluation reports.

## Abbreviations

The following abbreviations are used in this manuscript:

AI	Artificial Intelligence
BDI	Bile Duct Injury
CBD	Common Bile Duct
CV	Computer Vision
CVS	Critical View of Safety
IoU	Intersection over Union
LC	Laparoscopic Cholecystectomy
SANRA	Scale for the Assessment of Narrative Review Articles
STARD-AI	Standards for Reporting Diagnostic Accuracy Studies–Artificial Intelligence
