# Supplementary material for: Artificial Intelligence for Automated Recognition of Hepatocystic Anatomy During Laparoscopic Cholecystectomy: Current Evidence, Clinical Readiness, and Future Directions
Source: Medicina (Kaunas). 2026 Sep 5;62(9):1705. doi: 10.3390/medicina62091705 (PMC13609645; doi:10.3390/medicina62091705)
Supplement: Supplementary file 1 [file medicina-62-01705-s001.zip › medicina-4536472-supplementary.pdf]

Supplementary Table S1. Characteristics, validation design, principal performance, and translational status of 39 direct model-development or evaluation reports

**[18]**	Tokuyasu et al., 2021	Development: approximately 2000 images from 76 LC videos; separate test set of 23 videos.	YOLOv3 detection of the CBD, CD, liver segment IV edge, and Rouvière’s sulcus.	Video-level test set; two expert surgeons assessed overlay validity; one-case prototype verification. Bleeding and marked fibrosis or scarring were excluded from the development material.	AP: CBD, 0.320; CD, 0.074; segment IV edge, 0.314; and Rouvière’s sulcus, 0.101. Experts considered the indications valid in 22/23 test videos.	Stage 1. Separate-video technical validation; very limited live verification and restricted operative spectrum.
**[66]**	Inomata/Fujinaga et al., 2021	Development report from the linked Japanese navigation program; denominators for an independent evaluation were not provided.	Four-landmark anatomy-recognition concept and five-step development pathway for AI-navigated endoscopic surgery.	Technical and developmental description; no independent comparative test cohort or prespecified clinical endpoint.	Demonstrated the proposed system architecture and translational pathway; no independently interpretable performance estimate was reported.	Stage 0. Programmatic proof of concept.
**[21]**	Mascagni et al., 2021	EndoDigest: 155 LC videos comprising 55 development and 100 test videos.	Automatic localization of cystic-duct division and extraction of a 2.5-min CVS documentation clip.	Held-out video test set; human assessment of whether the automatically extracted clip permitted CVS evaluation.	Mean absolute localization error, 62.8 ± 130.4 s, corresponding to 1.95% of the full-video duration; the CVS was assessable in 91% of extracted clips.	Stage 1. Automated documentation support; not evidence of CVS achievement or improved safety outcomes.
**[48]**	Mascagni et al., 2022	A total of 174 LC videos from four centers; 144 videos were available for the primary analysis, including 27 fluorescence cases.	Multicenter evaluation of automatic cystic-duct-division localization and CVS clip extraction.	External multicenter video evaluation with expert assessment of documentation adequacy.	Mean localization error, 124.0 ± 270.6 s; 108/144 clips (75.0%) effectively documented the CVS.	Stage 2. Multicenter validation; performance was lower than in the initial development report.
**[22]**	Mascagni et al., 2022	A total of 2854 images from 201 LC videos; 402 images additionally received pixel-level annotations.	Deep-learning recognition of the three CVS criteria with associated anatomical segmentation.	Five-fold internal cross-validation; expert-derived frame labels and a subset of segmentation masks.	Segmentation mean IoU, 66.6%; CVS-criterion mAP, 71.9%; balanced accuracy, 71.4%.	Stage 1. Internal frame-level CVS assessment within the Endoscapes/EndoDigest research family.
**[19]**	Madani et al., 2022	A total of 2627 frames from 290 LC videos contributed by 153 surgeons at 136 institutions in 37 countries; four high-volume annotators.	Go/No-Go dissection-zone segmentation together with segmentation of the liver, gallbladder, and hepatocystic triangle.	Ten-fold internal cross-validation on a geographically broad but retrospectively sampled frame set.	Go zone: IoU, 0.53 ± 0.24; F1, 0.70 ± 0.28; sensitivity, 0.69; and specificity, 0.94. No-Go zone: IoU, 0.71 ± 0.29; F1, 0.83 ± 0.31; sensitivity, 0.80; and specificity, 0.98. Anatomical IoU: liver, 0.86; gallbladder, 0.72; and hepatocystic triangle, 0.65.	Stage 1. Broad source diversity, but no independent external cohort or live clinical comparison.
**[51]**	Colbeci et al., 2022	More than 2000 LC videos were used for video-based development of an attention multiple-instance-learning approach.	Recognition of individual CVS criteria and final CVS achievement from video.	Internal development and testing as reported; video-level labels rather than dense frame annotations.	Mean unweighted accuracy, 82.6% for individual CVS criteria and 84.2% for final CVS detection.	Stage 1. Promising weakly supervised video assessment; external reproducibility was not established.
**[55]**	Owen et al., 2022	A total of 3050 labeled and 3682 unlabeled LC frames.	Semi-supervised segmentation and presence detection of the cystic duct and cystic artery using label relaxation and pseudo-labeling.	Internal evaluation; inter-annotator IoU was reported, and the outputs were reviewed by three experts.	IoU, 65%; structure-presence F1, 75%; inter-annotator IoU, approximately 70%.	Stage 1. Annotation-efficient internal proof of performance.
**[24]**	Laplante et al., 2023	A total of 47 frames from 25 prospectively collected LC videos involving nine surgeons in three countries; six SAGES experts.	External validation of Go/No-Go zone segmentation.	Prospective, geographically separate sample with a six-expert reference assessment.	Go zone: F1/Dice, 0.58 ± 0.22; sensitivity, 0.52; and specificity, 0.97. No-Go zone: F1/Dice, 0.80 ± 0.12; sensitivity, 0.80; and specificity, 0.95. Accuracy was 0.92 for both classes.	Stage 2. Independent validation revealed materially lower sensitivity for the Go region.
**[26]**	Nakanuma et al., 2023	Prospective single-center J-SUMMIT-C-01 evaluation involving 10 LC	Real-time landmark-navigation overlay focused	EEC assessment comprising rubric scoring and evaluation of AI–expert	CBD landmark rubric score, 4.2 ± 0.8/5; AI–EEC Dice was reported as	Stage 3. Prospective intraoperative feasibility; very

		procedures.	on the CBD and other safety landmarks.	spatial agreement.	comparable with inter-EEC agreement.	small sample and no evaluation of operative or patient outcomes.
**[54]**	Fujinaga et al., 2023	Prospective J-SUMMIT-C-02 evaluation involving 20 LC procedures.	Real-time four-landmark navigation during the pre-dissection phase.	Expert review of phases in which landmark display was considered necessary and assessment of overlay quality.	Landmarks were detected in 92% of phases for which the EEC considered display necessary; mean rubric scores were 3.78 for the CBD and 3.67 for the CD. The rating of 3.65/4 for potential contribution to BDI prevention was subjective.	Stage 3. Live feasibility and expert acceptability; no observed BDI-prevention endpoint.
**[65]**	Endo et al., 2023	Twenty-second pre-dissection video segments were reviewed with and without AI by four beginner and four expert surgeons, producing 160 paired interpretation opportunities.	Effect of AI landmark overlays on operative-image interpretation and intended action.	Offline paired human-factor experiment with subsequent safety adjudication of changed responses.	AI changed 43/160 annotations (26.9%); 70% of these changes were judged to be safer.	Stage 1. Offline behavioral evidence rather than live intraoperative effectiveness.
**[23]**	Kawamura et al., 2023	A total of 72 LC videos yielding 23,793 labeled images.	Multi-landmark detection for real-time surgical navigation.	Internal video-model evaluation using reference annotations from the development program.	Best mean precision, 0.971; recall, 0.737; F1, 0.832; specificity, 0.966; and accuracy, 0.834. Processing speed was approximately 6 frames/s.	Stage 1. Near-real-time technical performance; no prospective comparative live evaluation.
**[20]**	Alkhamaiseh et al., 2023	A total of 1550 images sampled from 200 LC videos.	U-Net combined with an autoencoder for segmentation of LC anatomy.	Five-fold internal cross-validation.	Mean IoU, 74.65%; accuracy, 92.0%; and precision, 93.9%.	Stage 1. Internal image-level segmentation; generalizability and procedure-level independence require confirmation.
**[25]**	Khalid et al., 2023	Retrospective case–control analysis of 11 BDI videos and 11 cholecystitis controls without injury.	Application of GoNoGoNet to quantify the locations of sharp and blunt instrument interactions.	Video-based comparison of injury and control cases; retrospective association rather than prospective intervention.	Compared with controls, BDI cases had 23.5% more sharp interactions, 32.1% more blunt interactions, and 33.6% more total interactions outside the Go zone. Injury-causing interactions occurred in the No-Go zone in 4/11 cases, the Go zone in 2/11, and neither zone in 5/11.	Stage 2. Supports the construct validity of zone concepts but does not demonstrate that AI prevents BDI.
**[49]**	Wu et al., 2023	Multicenter development and application of SurgSmart; the quality application examined 431 LC videos.	Simultaneous recognition of operative phase, difficulty or severity, critical division, and the three CVS components.	Multicenter retrospective application with expert video assessment.	Accuracy: critical division, 98.49%; severity, 95.45%; phase, 88.61%; CVS I, 80.64%; CVS II, 97.62%; and CVS III, 78.87%. Complete CVS was documented in 4.33% of the 431 application videos.	Stage 2. Multicenter process and quality assessment; the low prevalence of complete CVS affects interpretation.
**[56]**	Smithmaitrie et al., 2024	A total of 3200 images obtained from 40 LC videos, with 80 images per video; annotations by three surgeons and an operating-room demonstration.	YOLOv7 detection of Rouvière’s sulcus and the base of liver segment IV to construct a guidance line.	Held-out image testing with expert acceptance assessment of the guidance line and a real-time operating-room demonstration.	Precision, 91.7%; recall, 86.5%; mAP, 91.3%; and processing time, 0.179 s/image. Both landmarks were detected in 342/360 relevant test images, and surgeons accepted the guidance line in 95.71%.	Stage 3. Demonstrated live use, but in a small selected series without a comparative clinical endpoint.
**[52]**	Petracchi et al., 2024	Prospective observational series of 40 consecutive elective LCs with blinded review by three surgeons.	YOLOv8-based recognition of CVS achievement.	Procedure-level comparison with expert assessment; difficult and non-elective cases were not represented.	AI detected CVS achievement in all 40 procedures and was reported to agree with expert assessment in 100% of cases.	Stage 2. Prospective validation within a narrow elective spectrum; no negative, complex-case, or patient-outcome evidence.
**[53]**	Leifman et al., 2024	Network trained using annotated segments from 700 videos; real-time operating-room validation in 40 consecutive elective LCs.	Live recognition of CVS achievement.	Surgeon reporting served as the procedure-level reference standard in the live cohort.	Surgeons reported CVS achievement in 34/40 cases, of which AI identified 33/34. All six cases without reported CVS achievement were classified as negative. Reported sensitivity was 97% (95% CI: 96.1–98.2), and	Stage 3. Live procedure-level feasibility; the reference standard and elective case mix limit effectiveness inference.

					specificity was 100% (95% CI: 98.1–100).	
**[60]**	Tashiro et al., 2024	Rapid preliminary clinical report; the exact evaluation denominator was not clearly available in the indexed report.	Real-time AI combined with ICG fluorescence to identify loose connective tissue and dissection planes.	Intraoperative demonstration with pixel-level comparison against expert-defined target tissue.	A Dice coefficient of 0.60 was subsequently reported for the preliminary system.	Stage 3*. Very early live demonstration with an insufficiently clear denominator and no comparative clinical endpoint.
**[28]**	Mascagni et al., 2024	SurgFlow was installed during three procedures for symptomatic cholelithiasis; eight stakeholders were interviewed.	Concurrent phase, instrument, anatomy, and CVS predictions from the operating-room video stream.	Prospective shadow-mode technical feasibility; predictions used a secondary signal and were not displayed to the operating surgeon.	No system malfunctions were reported during the three procedures; stakeholder interviews identified workflow and deployment considerations.	Stage 3. Operating-room feasibility in shadow mode rather than surgeon-facing efficacy.
**[67]**	Murali et al., 2024	Endoscapes+: 11,090 images from 201 videos, including 1933 spatially annotated images; official 120/41/40-video split.	Label-efficient graph-based CVS recognition using bounding boxes or masks.	Procedure- or video-level held-out benchmark testing within Endoscapes.	LG-CVS mAP, 63.6 with bounding boxes and 67.3 with masks; DeepCVS baseline mAP, 62.8.	Stage 1. Benchmark advancement within a recurrent public-dataset family.
**[64]**	Ghobadi et al., 2024	A total of 4040 CholecSeg8k images from 17 videos; random 2828/808/404-image split and additional unlabeled private data.	FPN with Inception-ResNet-v2 for semantic segmentation of surgical anatomy.	High internal image-level performance; qualitative independent-domain assessment because the private images lacked labels.	Internal mean IoU, 0.95896; F1, 0.9773. Visual review of the private dataset revealed substantial false-negative segmentation, precluding an external quantitative estimate.	Stage 2. External-transfer attempt revealed domain-shift failure despite exceptional internal metrics.
**[62]**	Orimoto et al., 2025	Development: 2025 images from 21 acute-cholecystitis cases; external EEC assessment involving 20 cases from other centers.	Detection and segmentation of scar tissue in acute cholecystitis.	External multicenter expert evaluation.	Scar-tissue Dice, 0.612; mean expert detection score, 3.98 ± 0.76/5, with perceived clinical and educational utility.	Stage 2. Addresses a difficult operative domain; modest overlap and subjective utility require prospective confirmation.
**[63]**	Tokuyasu et al., 2025	A total of 99 bleeding-field images from 20 patients, annotated by three board-certified surgeons.	Bleeding-robust landmark recognition using CycleGAN-generated pseudo-nonbleeding images and uncertainty fusion.	Internal comparison with a conventional model, supplemented by expert visibility and boundary assessment.	Dice improved significantly for all three annotator comparisons ($p < 0.001$); mean processing time was 0.06 s/frame using an RTX A5000. A pooled absolute Dice value was unavailable in the indexed report.	Stage 1. Technical robustness experiment using a small dataset; not evidence of calibration or live safety.
**[59]**	Yin et al., 2025	A total of 113 ICG-guided LCs and 3993 selected images; approximate patient-level 8:1:1 split and short-video validation.	Six-class YOLOv7 anatomical detection during fluorescence cholangiography.	Internal patient-level testing and short-video analysis; an intraoperative prototype was developed, but no formal live user-effect cohort was reported.	Overall mAP, 0.846; AP: CBD, 0.864, and CD, 0.698. Video accuracy: CBD, 94.39%, and CD, 84.97%; sensitivity, 99.36% and 87.98%; specificity, 19.35% and 88.89%; and F1, 97.08% and 85.92%, respectively. CBD-positive prevalence in the short-video set was 468/499 clips (93.79%; inferred from the reported confusion matrix).	Stage 1. Strong detection signal, but very low CBD specificity illustrates prevalence and threshold dependence; high-difficulty cases were underrepresented.
**[61]**	Hou et al., 2025	A total of 200 LC videos and 3796 ICG-overlay images, comprising 2979 training and 817 validation images.	YOLOv5s bounding-box detection of the hepatocystic triangle on fluorescence-overlay images.	Internal image-level development and validation; procedure-level independence was not clearly reported.	Optimal non-maximum-suppression IoU threshold, 0.6; AP, 0.859. Higher thresholds of 0.7–0.9 increased duplicate predictions.	Stage 0. Proof of concept with unclear independence of image partitions and no held-out clinical test cohort.
**[74]**	Yang et al., 2025	A total of 1425 frames from 132 LC videos; ten-fold evaluation using pre-dissection frames for training and post-dissection frames for testing.	CPPN semantic segmentation of the gallbladder, hepatocystic or Calot region, and CBD.	Internal phase-based evaluation; potential dependence involving different phases of the same procedure was not clearly excluded.	Mean IoU, 0.855 ± 0.03; class-specific IoU: gallbladder, 0.881 ± 0.01; hepatocystic or Calot region, 0.769 ± 0.03; and CBD, 0.813 ± 0.02.	Stage 0. High internal segmentation values, but the independence of the data split was insufficiently clear.
**[71]**	Long et al., 2025	Cholec80: 544 labeled images from three videos, comprising 323 training, 133 validation, and 88 test images,	Lightweight YOLO and semi-supervised LC-YOLOmatch anatomical	Internal public-dataset testing; labeled test diversity was limited to three source videos.	LC-YOLO performance on 88 test images: precision, 0.586; recall, 0.712; F1, 0.633; mAP50, 0.696; and	Stage 1. Efficient benchmarking; limited labeled-video diversity constrains

		plus 2550 unlabeled images from 77 videos.	detection.		mAP50–95, 0.364. Semi-supervised LC-YOLOmatch achieved an mAP50 of 70.0% and an mAP50–95 of 40.8%.	generalizability.
**[72]**	Xu et al., 2025	Public Endoscapes-BBox201 dataset comprising 1933 frames with an official 1212/409/312 split, together with the m2cai16-tool-locations benchmark.	Relation-LCSOD relational object detection of anatomical structures and instruments.	Official benchmark comparisons against contemporary detectors.	Overall mAP improved by 33.95% relative to the strongest comparator on Endoscapes; reported relative AP gains included 90.32% for the cystic plate and 92.46% for the hepatocystic triangle. Nevertheless, absolute AP50–95 remained below 25% for the cystic plate, cystic artery, and cystic duct across models.	Stage 1. Relative gains did not eliminate poor performance involving small safety-critical structures.
**[68]**	Nowak et al., 2025	Endoscapes2023: 201 videos, 58,585 frames, 11,090 CVS labels, and 493 masks; official video-level split.	Temporal SwinCVS models for frame- and video-aware CVS recognition.	Held-out benchmark testing with procedure- or video-level separation.	SwinV2 frame mAP, 64.52%; frozen temporal SwinCVS mAP, 67.45%; and balanced accuracy, 70.25%. End-to-end mAP was 64.59% versus 64.68% for SV2LSTG ($p = 0.470$); latency was 107.8 ± 8 ms/frame, corresponding to approximately 9 Hz.	Stage 1. Transparent temporal benchmark; modest absolute gains and no external clinical cohort.
**[75]**	Guo et al., 2026	Robotic MT-CVS dataset comprising 106 procedures, 4860 frames, and 965 paired masks; procedure-level 7:1:2 split and additional Endoscapes evaluation.	Dual-task meta-auxiliary learning for CVS classification and anatomical segmentation.	Internal procedure-level evaluation supplemented by public-benchmark testing.	CVS F1, 78%; segmentation mean IoU, 83.9%. On MT-CVS, anatomical segmentation improved by more than 14.6 percentage points in IoU and 13.1 points in Dice relative to comparator models.	Stage 1. Strong internal dual-task results; robotic modality and benchmark reuse limit direct inference regarding conventional LC.
**[69]**	Wang et al., 2026	Endoscapes2023: 201 videos and approximately 58,813 frames; official 120/41/40-video split.	SMIL label-free distillation and multiple-instance learning for CVS recognition.	Held-out Endoscapes benchmark with comparisons against label-free and segmentation-supervised methods.	Compared with the strongest label-free baseline, mAP improved by 3.21 percentage points and balanced accuracy by 2.74 percentage points; the model exceeded segmentation-supervised comparators in mAP. Absolute values were unavailable in the indexed preview used for extraction.	Stage 1. Annotation-efficient benchmark result; recurrent dataset family and incomplete indexed numerical detail.
**[50]**	M. Tang et al., 2026	A total of 377 LC videos from 17 hospitals for development, validation, and testing; deployed at three hospitals from May to October 2024 with 18 surgeons.	SurgSmart CVS-component scoring and quality-improvement feedback.	Multicenter development followed by post-deployment process surveillance.	Accuracy/macro-F1: CVS I, 0.91/0.72; CVS II, 0.86/0.67; and CVS III, 0.73/0.70. Overall CVS scores improved following deployment ($p < 0.01$); 15/18 surgeons improved ($p < 0.05$), with high reported satisfaction.	Stage 4A†. Post-deployment process evidence only; no comparator-controlled patient-level safety or effectiveness endpoint.
**[57]**	K. Tang et al., 2026	A total of 186 single-incision LC videos from two institutions; 127 videos and 6650 frames for development and 59 videos and 2999 frames for testing; three expert annotators.	Mask2Former segmentation of safe and hazardous dissection regions during single-incision LC.	Internal two-institution held-out video evaluation.	Safe region: F1, 0.966; IoU, 0.805; sensitivity, 0.968; and PPV, 0.942. Hazard region: F1, 0.923; Dice, 0.825; IoU, 0.770; and sensitivity, 0.970.	Stage 1. Strong held-out performance within a specific operative approach; no live user or external-platform evaluation.
**[58]**	Sonoda et al., 2026	DeepLabv3+ developed using 73 videos; external testing using 10 independent videos and 100 frames; randomized video-based pilot involving ten postgraduate-year-5/6 surgeons and 20 tasks each.	Segmentation of an alert zone and gallbladder surface combined with AI-assisted incision-site selection.	Independent expert ground truth and randomized simulated human-factor comparison.	IoU against developer annotations: alert zone, 0.703, and gallbladder, 0.735; against external ground truth: 0.706 and 0.730, respectively. Correct safe-incision selection improved from 58% to 90%, and gallbladder-contour recognition improved from 70% to 92% (both $p < 0.05$).	Stage 2. External technical and simulated behavioral evidence; not a prospective live operating-room trial.

**[73]**	Yang et al., 2024 (conference)	Peer-reviewed conference report; detailed cohort denominators were unavailable in the indexed record.	Improved YOLOv7 with channel attention for landmark detection during LC.	Internal technical comparison without independent clinical validation.	The conference report described improved mAP, precision, and recall relative to the comparator; absolute values could not be verified from the indexed summary.	Stage 0. Preliminary technical evidence requiring complete denominator reporting and external verification.
**[76]**	Zhou et al., 2024 (conference)	Peer-reviewed ISBI report; detailed denominators were unavailable in the indexed record.	Boundary-fuzziness method for surgical region-of-interest detection.	Internal technical evaluation; no external or clinical validation was reported in the indexed material.	Technical feasibility was reported; an independently extractable clinical-performance estimate was unavailable.	Stage 0. Preliminary technical report.
**[70]**	Li et al., 2024 (conference)	A total of 5750 annotated LC frames.	Object detection combined with the Segment Anything Model for CVS assessment.	Internal frame- and video-level evaluation; the comparator included LG-CVS within the broader Endoscapes research ecosystem.	Competitive frame-level performance and an approximately 20% improvement in video-level CVS assessment relative to LG-CVS were reported.	Stage 1. Internal benchmark advancement; external clinical validation was absent.

Notes: Values are reproduced at the unit and aggregation level reported by each study and should not be compared across dissimilar targets. Stage 0 = offline proof of concept; Stage 1 = internal held-out or cross-validated evaluation with appropriate patient- or procedure-level separation; Stage 2 = temporal, external, or multicenter validation; Stage 3 = prospective real-time feasibility or operating-room human-factor evaluation; Stage 4A = post-deployment surveillance of technical reliability, human interaction, or operative-process outcomes without comparator-controlled patient-level effectiveness; and Stage 4B = comparative clinical-effectiveness evidence demonstrating patient-level safety or benefit. Reference [75] was classified as Stage 3 because live intraoperative use was described, although the evidence was preliminary and the evaluation denominator was insufficiently clear. Reference [59] reached Stage 4A solely through post-deployment process surveillance; no patient-level safety or effectiveness endpoint was evaluated. No report met Stage 4B criteria. ‘Not available in the indexed report’ indicates that an exact value or denominator could not be verified and that no value was inferred.

Abbreviations: AP, average precision; BDI, bile duct injury; CBD, common bile duct; CD, cystic duct; CI, confidence interval; CVS, critical view of safety; EEC, external expert-evaluation committee; F1, harmonic mean of precision and recall; FPN, feature pyramid network; ICG, indocyanine green; IoU, intersection-over-union; LC, laparoscopic cholecystectomy; mAP, mean average precision; PPV, positive predictive value; SAGES, Society of American Gastrointestinal and Endoscopic Surgeons.
